# The galla[1]ferrocenophane {[dimeth­yl(2-pyrid­yl)sil­yl]bis­(trimethyl­silyl)methyl-κ^2^ *C*,*N*}(ferrocene-1,1′-di­yl)gallium(III)

**DOI:** 10.1107/S1600536808005503

**Published:** 2008-03-05

**Authors:** Jörg A. Schachner, J. Wilson Quail, Jens Müller

**Affiliations:** a Department of Chemistry, University of Saskatchewan, 110 Science Place, Saskatoon, Saskatchewan, Canada S7N 5C9; b Saskatchewan Structural Sciences Centre, University of Saskatchewan, 110 Science Place, Saskatoon, Saskatchewan, Canada S7N 5C9

## Abstract

The title compound, [GaFe(C_5_H_4_)_2_(C_14_H_28_NSi_3_)] or [{(2-H_4_C_5_N)Me_2_Si}(Me_3_Si)_2_C]Ga(C_5_H_4_)_2_Fe, a galla[1]ferrocenophane, crystallizes with two independent mol­ecules in the asymmetric unit. In these strained sandwich compounds, the angles between the planes of the two π-ligands are 15.4 (2) and 16.4 (2)°, with gallium in a distorted tetrahedral coordination environment.

## Related literature

The synthesis of the title compound was described by Schachner *et al.* (2005*b* ▶). A related galla[1]ferrocenophane was published by Lund *et al.* (2006 ▶). For related literature, see: Bellas & Rehahn (2007 ▶); Foucher *et al.* (1992 ▶); Herbert *et al.* (2007 ▶); Lund *et al.* (2007 ▶); Osborne & Whiteley (1975 ▶); Schachner *et al.* (2005*a* ▶, 2007 ▶).
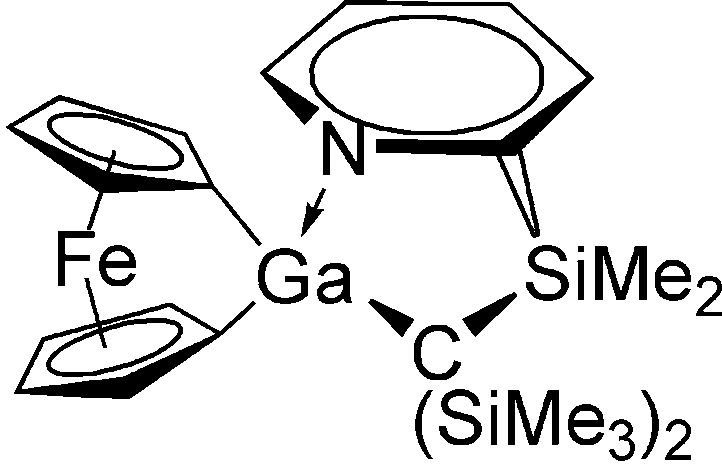


## Experimental

### 

#### Crystal data

[GaFe(C_5_H_4_)_2_(C_14_H_28_NSi_3_)]
*M* *_r_* = 548.38
Monoclinic, 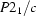

*a* = 9.64630 (10) Å
*b* = 35.2258 (4) Å
*c* = 15.4862 (2) Å
β = 92.5212 (7)°
*V* = 5257.10 (11) Å^3^
*Z* = 8
Mo *K*α radiation
μ = 1.73 mm^−1^
*T* = 173 (2) K
0.20 × 0.12 × 0.12 mm

#### Data collection

Nonius KappaCCD diffractometer
Absorption correction: ψ scan (*SHELXTL*; Sheldrick, 2008 ▶) *T*_min_ = 0.692, *T*_max_ = 0.811
58270 measured reflections
10370 independent reflections
7767 reflections with *I* > 2σ(*I*)
*R*_int_ = 0.079

#### Refinement

*R*[*F* ^2^ > 2σ(*F* ^2^)] = 0.043
*wR*(*F* ^2^) = 0.088
*S* = 1.04
10370 reflections
557 parameters
H-atom parameters constrained
Δρ_max_ = 0.36 e Å^−3^
Δρ_min_ = −0.46 e Å^−3^

### 

Data collection: *COLLECT* (Nonius, 1998 ▶); cell refinement: *SCALEPACK* (Otwinowski & Minor, 1997 ▶); data reduction: *SCALEPACK* and *DENZO* (Otwinowski & Minor, 1997 ▶); program(s) used to solve structure: *SIR97* (Altomare *et al*., 1999 ▶); program(s) used to refine structure: *SHELXL97* (Sheldrick, 2008 ▶); molecular graphics: *ORTEP-3 for Windows* (Farrugia, 1997 ▶); software used to prepare material for publication: *SHELXL97* and *PLATON* (Spek, 2003 ▶).

## Supplementary Material

Crystal structure: contains datablocks I, global. DOI: 10.1107/S1600536808005503/om2215sup1.cif

Structure factors: contains datablocks I. DOI: 10.1107/S1600536808005503/om2215Isup2.hkl

Additional supplementary materials:  crystallographic information; 3D view; checkCIF report

## Figures and Tables

**Fig. 1.**
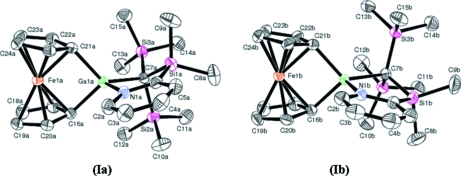
Plot of the two independent molecules of with thermal ellipsoids at the 50% probability level. H atoms are omitted for claritity.

**Fig. 2.**
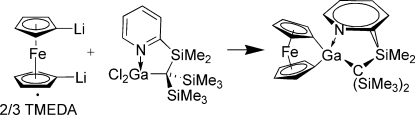
The formation of the title compound.

## supplementary crystallographic information

### Comment

After the first report of the synthesis of a [1]ferrocenophane by Osborne and Whiteley in 1975 (Osborne *et al.*, 1975), Manners and coworkers discovered that thermal ring-opening polymerization (ROP) of dimethylsila[1]ferrocenophane yield high-molecular weight polymers (Foucher *et al.*, 1992). This discovery was a breakthrough in the area of organometallic polymers. Recently, the field of strained sandwich compounds was reviewed by Manners (Herbert *et al.*, 2007) and Rehahn (Bellas *et al.*, 2007).

Over the last four years, we characterized aluminium- and gallium-bridged [1]ferrocenophanes (Schachner *et al.*, 2005*a*, b), (Lund *et al.*, 2006), [1]chromarenophanes (Lund *et al.*, 2006), [1]vanadarenophanes (Lund *et al.*, 2006), [1]ruthenocenophanes (Schachner *et al.*, 2007), and [1]molybdarenophanes (Lund *et al.*, 2007).

We published the synthesis and characterization of the first strained sandwich compound with gallium in the bridging position in 2005 (Schachner *et al.*, 2005*b*) (Scheme 1). However, details of the molecular structure of the title compound could not be extracted from the single-crystal X-ray analysis; only the molecular framework was revealed with certainty from the analysis (Schachner *et al.*, 2005*b*). After several attempts, we succeeded in growing single crystals of high quality that allowed a complete structural solution of the compound. The galla[1]ferrocenophane crystallizes in the monoclinic space group *P*2_1_/*c* with two independent molecules in the asymmetric unit. Figure 1 depicts the two independent species. In both molecules the bond lengths around the bridging Ga atoms are very similar (Ga1a—C16*a* = 2.018 (3); Ga1b—C16*b* = 2.026 (3); Ga1a—C21*a* = 2.023 (3); Ga1b—C21*b* = 2.023 (3); Ga1a—N1a = 2.084 (3); Ga1b—N1b = 2.050 (3); Ga1a—C7a = 2.043 (3); Ga1b—C7b = 2.038 (3) Å). [1]Metallacyclophanes can only be ring-open polymerized because they are intrinsically strained. The so-called α angle in strained sandwich compounds is defined as the angle between the two planes of two π-ligands. The angle between the two least squares planes of C16 - C20 and C21 - C25 are 15.4 (2)° for molecule (Ia) and 16.4 (2)° for molecule (Ib). Both values compare well with the value of the 15.83 (19)° found for the only other known galla[1]ferrocenophane (Lund *et al.*, 2006).

### Experimental

The title compound was synthesized as described in the literature (Schachner *et al.* 2005*b*). Single crystals were obtained by crystallization from a hexane solution at *ca* -25°C.

### Refinement

H atoms were placed in calculated positions with C—H = 0.95Å and *U*_iso_ constrained to be 1.2 times *U*_eq_ of the carrier atom for aromatic protons and C—H = 0.98Å with *U*_iso_ constrained to 1.5 times *U*_eq_ of the carrier atom for methyl hydrogen atoms.

### Crystal data

**Table:**

[GaFe(C_5_H_4_)_2_(C_14_H_28_NSi_3_)]	*F*_000_ = 2288
*M**_r_* = 548.38	*D*_x_ = 1.386 Mg m^−^^3^
Monoclinic, *P*2_1_/*c*	Mo *K*α radiation λ = 0.71073 Å
Hall symbol: -P 2ybc	Cell parameters from 91449 reflections
*a* = 9.64630 (10) Å	θ = 1.0–26.0º
*b* = 35.2258 (4) Å	µ = 1.73 mm^−^^1^
*c* = 15.4862 (2) Å	*T* = 173 (2) K
β = 92.5212 (7)º	Chip, orange
*V* = 5257.10 (11) Å^3^	0.20 × 0.12 × 0.12 mm
*Z* = 8	

### Data collection

**Table:**

Nonius KappaCCD diffractometer	10370 independent reflections
Radiation source: fine-focus sealed tube	7767 reflections with *I* > 2σ(*I*)
Monochromator: horizonally mounted graphite crystal	*R*_int_ = 0.079
Detector resolution: 9 pixels mm^-1^	θ_max_ = 26.1º
*T* = 173(2) K	θ_min_ = 1.4º
φ scans, and ω scans with κ offsets	*h* = −11→11
Absorption correction: ψ scan(SHELXTL; Sheldrick, 2008)	*k* = −43→43
*T*_min_ = 0.692, *T*_max_ = 0.811	*l* = −19→19
58270 measured reflections	

### Refinement

**Table:**

Refinement on *F*^2^	Secondary atom site location: difference Fourier map
Least-squares matrix: full	Hydrogen site location: inferred from neighbouring sites
*R*[*F*^2^ > 2σ(*F*^2^)] = 0.043	H-atom parameters constrained
*wR*(*F*^2^) = 0.088	*w* = 1/[σ^2^(*F*_o_^2^) + (0.0238*P*)^2^ + 7.0617*P*] where *P* = (*F*_o_^2^ + 2*F*_c_^2^)/3
*S* = 1.04	(Δ/σ)_max_ = 0.001
10370 reflections	Δρ_max_ = 0.36 e Å^−^^3^
557 parameters	Δρ_min_ = −0.45 e Å^−^^3^
Primary atom site location: structure-invariant direct methods	Extinction correction: none

### Special details

**Table:**

Geometry. All e.s.d.'s (except the e.s.d. in the dihedral angle between two l.s. planes) are estimated using the full covariance matrix. The cell e.s.d.'s are taken into account individually in the estimation of e.s.d.'s in distances, angles and torsion angles; correlations between e.s.d.'s in cell parameters are only used when they are defined by crystal symmetry. An approximate (isotropic) treatment of cell e.s.d.'s is used for estimating e.s.d.'s involving l.s. planes.
Refinement. Refinement of *F*^2^ against ALL reflections. The weighted *R*-factor *wR* and goodness of fit *S* are based on *F*^2^, conventional *R*-factors *R* are based on *F*, with *F* set to zero for negative *F*^2^. The threshold expression of *F*^2^ > σ(*F*^2^) is used only for calculating *R*-factors(gt) *etc*. and is not relevant to the choice of reflections for refinement. *R*-factors based on *F*^2^ are statistically about twice as large as those based on *F*, and *R*- factors based on ALL data will be even larger.

### Fractional atomic coordinates and isotropic or equivalent isotropic displacement parameters (Å^2^)

**Table:**

	*x*	*y*	*z*	*U*_iso_*/*U*_eq_	
Fe1A	0.30497 (5)	0.055334 (13)	1.00300 (3)	0.02692 (12)	
Ga1A	0.14191 (4)	0.116989 (10)	0.94201 (2)	0.02454 (10)	
N1A	0.0812 (3)	0.11264 (7)	0.81155 (17)	0.0257 (6)	
Si1A	0.05479 (11)	0.18896 (3)	0.85081 (6)	0.0299 (2)	
Si2A	−0.13709 (10)	0.15670 (3)	0.98932 (7)	0.0317 (2)	
Si3A	0.14012 (11)	0.19919 (3)	1.04473 (7)	0.0317 (2)	
C2A	0.0799 (4)	0.08122 (10)	0.7626 (2)	0.0334 (9)	
H2A	0.1057	0.0578	0.7889	0.040*	
C3A	0.0429 (4)	0.08158 (11)	0.6762 (2)	0.0379 (9)	
H3A	0.0420	0.0587	0.6437	0.045*	
C4A	0.0070 (4)	0.11542 (11)	0.6368 (2)	0.0374 (9)	
H4A	−0.0184	0.1164	0.5769	0.045*	
C5A	0.0090 (4)	0.14802 (10)	0.6872 (2)	0.0343 (9)	
H5A	−0.0148	0.1717	0.6613	0.041*	
C6A	0.0454 (3)	0.14648 (9)	0.7748 (2)	0.0275 (8)	
C7A	0.0484 (3)	0.16817 (9)	0.9611 (2)	0.0255 (8)	
C8A	−0.0866 (5)	0.22219 (11)	0.8131 (3)	0.0520 (12)	
H8A	−0.1767	0.2096	0.8171	0.078*	
H8B	−0.0730	0.2294	0.7530	0.078*	
H8C	−0.0842	0.2449	0.8496	0.078*	
C9A	0.2214 (5)	0.21208 (12)	0.8242 (3)	0.0541 (12)	
H9A	0.2313	0.2361	0.8558	0.081*	
H9B	0.2215	0.2170	0.7619	0.081*	
H9C	0.2990	0.1953	0.8410	0.081*	
C10A	−0.2308 (4)	0.13154 (13)	0.8976 (3)	0.0521 (12)	
H10A	−0.3264	0.1263	0.9130	0.078*	
H10B	−0.1835	0.1076	0.8861	0.078*	
H10C	−0.2315	0.1476	0.8459	0.078*	
C11A	−0.2409 (4)	0.19977 (11)	1.0156 (3)	0.0498 (11)	
H11A	−0.3387	0.1927	1.0191	0.075*	
H11B	−0.2314	0.2189	0.9703	0.075*	
H11C	−0.2067	0.2102	1.0712	0.075*	
C12A	−0.1518 (4)	0.12448 (11)	1.0844 (3)	0.0467 (11)	
H12A	−0.1044	0.1360	1.1352	0.070*	
H12B	−0.1088	0.1000	1.0720	0.070*	
H12C	−0.2499	0.1206	1.0957	0.070*	
C13A	0.1152 (5)	0.18302 (12)	1.1584 (2)	0.0528 (12)	
H13A	0.1377	0.1560	1.1634	0.079*	
H13B	0.0184	0.1871	1.1728	0.079*	
H13C	0.1764	0.1976	1.1982	0.079*	
C14A	0.0752 (4)	0.24962 (10)	1.0403 (3)	0.0466 (11)	
H14A	0.1308	0.2652	1.0812	0.070*	
H14B	−0.0222	0.2503	1.0556	0.070*	
H14C	0.0835	0.2596	0.9817	0.070*	
C15A	0.3327 (4)	0.20227 (11)	1.0337 (3)	0.0474 (11)	
H15A	0.3765	0.2120	1.0875	0.071*	
H15B	0.3528	0.2194	0.9860	0.071*	
H15C	0.3696	0.1770	1.0217	0.071*	
C16A	0.0984 (3)	0.06751 (9)	1.0006 (2)	0.0298 (8)	
C17A	0.1548 (4)	0.06341 (11)	1.0879 (2)	0.0368 (9)	
H17A	0.1535	0.0826	1.1311	0.044*	
C18A	0.2125 (4)	0.02665 (11)	1.1002 (3)	0.0431 (10)	
H18A	0.2573	0.0173	1.1518	0.052*	
C19A	0.1917 (4)	0.00647 (11)	1.0221 (3)	0.0418 (10)	
H19A	0.2191	−0.0190	1.0117	0.050*	
C20A	0.1222 (4)	0.03111 (10)	0.9615 (3)	0.0344 (9)	
H20A	0.0953	0.0245	0.9037	0.041*	
C21A	0.3479 (3)	0.10609 (9)	0.9457 (2)	0.0273 (8)	
C22A	0.4259 (4)	0.10155 (10)	1.0276 (2)	0.0317 (8)	
H22A	0.4206	0.1182	1.0756	0.038*	
C23A	0.5110 (4)	0.06870 (10)	1.0254 (2)	0.0341 (9)	
H23A	0.5710	0.0597	1.0712	0.041*	
C24A	0.4909 (4)	0.05174 (10)	0.9432 (2)	0.0323 (8)	
H24A	0.5345	0.0293	0.9237	0.039*	
C25A	0.3930 (4)	0.07447 (9)	0.8946 (2)	0.0280 (8)	
H25A	0.3617	0.0695	0.8367	0.034*	
Fe1B	0.77578 (5)	0.031639 (13)	0.33294 (3)	0.02690 (12)	
Ga1B	0.63939 (4)	0.087176 (10)	0.43069 (2)	0.02319 (9)	
N1b	0.6415 (3)	0.07546 (8)	0.56039 (18)	0.0275 (7)	
Si1B	0.55460 (11)	0.15070 (3)	0.55006 (7)	0.0372 (3)	
Si2B	0.59969 (11)	0.17257 (3)	0.35803 (7)	0.0360 (3)	
Si3B	0.34124 (10)	0.12263 (3)	0.41012 (7)	0.0292 (2)	
C2B	0.6827 (4)	0.04260 (10)	0.5972 (2)	0.0333 (9)	
H2B	0.7100	0.0224	0.5610	0.040*	
C3B	0.6869 (4)	0.03707 (11)	0.6852 (2)	0.0401 (10)	
H3B	0.7174	0.0136	0.7094	0.048*	
C4B	0.6463 (4)	0.06618 (13)	0.7373 (3)	0.0443 (10)	
H4B	0.6487	0.0631	0.7983	0.053*	
C5B	0.6016 (4)	0.10014 (12)	0.6998 (2)	0.0423 (10)	
H5B	0.5722	0.1204	0.7353	0.051*	
C6B	0.5999 (4)	0.10457 (10)	0.6105 (2)	0.0325 (8)	
C7B	0.5295 (4)	0.13650 (9)	0.4346 (2)	0.0281 (8)	
C8B	0.7064 (5)	0.18256 (12)	0.5764 (3)	0.0607 (13)	
H8D	0.7896	0.1721	0.5514	0.091*	
H8E	0.7211	0.1844	0.6393	0.091*	
H8F	0.6877	0.2079	0.5524	0.091*	
C9B	0.4067 (5)	0.17341 (14)	0.6057 (3)	0.0661 (15)	
H9D	0.3848	0.1979	0.5786	0.099*	
H9E	0.4330	0.1773	0.6669	0.099*	
H9F	0.3251	0.1568	0.6006	0.099*	
C10B	0.7926 (4)	0.17796 (11)	0.3689 (3)	0.0554 (13)	
H10D	0.8165	0.1963	0.4147	0.083*	
H10E	0.8275	0.1871	0.3141	0.083*	
H10F	0.8349	0.1534	0.3834	0.083*	
C11B	0.5249 (5)	0.22135 (10)	0.3737 (3)	0.0552 (12)	
H11D	0.5395	0.2290	0.4343	0.083*	
H11E	0.4253	0.2210	0.3584	0.083*	
H11F	0.5711	0.2395	0.3365	0.083*	
C12B	0.5588 (5)	0.16013 (12)	0.2420 (3)	0.0544 (12)	
H12D	0.6085	0.1774	0.2046	0.082*	
H12E	0.4587	0.1625	0.2295	0.082*	
H12F	0.5879	0.1340	0.2312	0.082*	
C13B	0.3019 (4)	0.09920 (11)	0.3038 (2)	0.0414 (10)	
H13D	0.3488	0.0745	0.3026	0.062*	
H13E	0.3348	0.1153	0.2573	0.062*	
H13F	0.2015	0.0955	0.2958	0.062*	
C14B	0.2193 (4)	0.16437 (12)	0.4105 (3)	0.0508 (11)	
H14D	0.1234	0.1552	0.4104	0.076*	
H14E	0.2319	0.1799	0.3588	0.076*	
H14F	0.2393	0.1797	0.4623	0.076*	
C15B	0.2855 (4)	0.08709 (12)	0.4914 (3)	0.0465 (10)	
H15D	0.1868	0.0813	0.4809	0.070*	
H15E	0.3005	0.0975	0.5497	0.070*	
H15F	0.3401	0.0638	0.4861	0.070*	
C16B	0.8391 (3)	0.07929 (9)	0.3993 (2)	0.0290 (8)	
C17B	0.8717 (4)	0.08150 (10)	0.3093 (2)	0.0364 (9)	
H17B	0.8392	0.1007	0.2702	0.044*	
C18B	0.9598 (4)	0.05080 (11)	0.2876 (3)	0.0432 (10)	
H18B	0.9946	0.0459	0.2323	0.052*	
C19B	0.9862 (4)	0.02905 (10)	0.3628 (3)	0.0375 (9)	
H19B	1.0426	0.0070	0.3673	0.045*	
C20B	0.9137 (3)	0.04586 (10)	0.4309 (2)	0.0311 (8)	
H20B	0.9140	0.0366	0.4886	0.037*	
C21B	0.5751 (3)	0.04071 (9)	0.3634 (2)	0.0278 (8)	
C22B	0.5895 (4)	0.04133 (10)	0.2716 (2)	0.0325 (8)	
H22B	0.5675	0.0624	0.2353	0.039*	
C23B	0.6413 (4)	0.00605 (10)	0.2423 (2)	0.0367 (9)	
H23B	0.6596	−0.0005	0.1844	0.044*	
C24B	0.6603 (4)	−0.01748 (10)	0.3161 (2)	0.0332 (9)	
H24B	0.6943	−0.0428	0.3165	0.040*	
C25B	0.6200 (3)	0.00323 (9)	0.3893 (2)	0.0277 (8)	
H25B	0.6222	−0.0062	0.4468	0.033*	

### Atomic displacement parameters (Å^2^)

**Table:**

	*U*^11^	*U*^22^	*U*^33^	*U*^12^	*U*^13^	*U*^23^
Fe1A	0.0237 (3)	0.0279 (3)	0.0293 (3)	0.0008 (2)	0.0036 (2)	0.0040 (2)
Ga1A	0.0245 (2)	0.02234 (19)	0.0269 (2)	0.00140 (16)	0.00234 (16)	0.00005 (16)
N1A	0.0240 (16)	0.0248 (15)	0.0285 (16)	0.0033 (12)	0.0020 (13)	−0.0026 (12)
Si1A	0.0384 (6)	0.0232 (5)	0.0281 (6)	0.0002 (4)	0.0015 (5)	0.0006 (4)
Si2A	0.0253 (6)	0.0349 (6)	0.0352 (6)	0.0012 (4)	0.0047 (4)	−0.0018 (5)
Si3A	0.0360 (6)	0.0266 (5)	0.0321 (6)	0.0001 (4)	−0.0032 (5)	−0.0039 (4)
C2A	0.038 (2)	0.0275 (19)	0.035 (2)	0.0055 (16)	−0.0009 (17)	−0.0054 (16)
C3A	0.040 (2)	0.037 (2)	0.037 (2)	0.0049 (18)	−0.0002 (18)	−0.0118 (18)
C4A	0.035 (2)	0.051 (2)	0.026 (2)	0.0050 (18)	−0.0028 (17)	−0.0065 (18)
C5A	0.034 (2)	0.036 (2)	0.033 (2)	0.0057 (17)	0.0007 (17)	0.0033 (17)
C6A	0.0212 (18)	0.0318 (19)	0.030 (2)	0.0002 (15)	0.0021 (15)	0.0006 (15)
C7A	0.0252 (19)	0.0224 (17)	0.0289 (19)	−0.0001 (14)	0.0019 (15)	−0.0025 (14)
C8A	0.079 (3)	0.037 (2)	0.040 (3)	0.020 (2)	−0.005 (2)	0.0018 (19)
C9A	0.071 (3)	0.053 (3)	0.039 (3)	−0.027 (2)	0.012 (2)	−0.002 (2)
C10A	0.032 (2)	0.067 (3)	0.058 (3)	−0.011 (2)	0.003 (2)	−0.013 (2)
C11A	0.035 (2)	0.054 (3)	0.061 (3)	0.012 (2)	0.009 (2)	−0.002 (2)
C12A	0.040 (2)	0.050 (3)	0.051 (3)	0.000 (2)	0.014 (2)	0.005 (2)
C13A	0.074 (3)	0.053 (3)	0.031 (2)	−0.007 (2)	−0.011 (2)	−0.004 (2)
C14A	0.054 (3)	0.028 (2)	0.057 (3)	−0.0011 (19)	0.000 (2)	−0.0091 (19)
C15A	0.039 (2)	0.042 (2)	0.060 (3)	−0.0063 (19)	−0.006 (2)	−0.013 (2)
C16A	0.0224 (19)	0.0279 (19)	0.040 (2)	0.0024 (15)	0.0076 (16)	0.0037 (16)
C17A	0.037 (2)	0.041 (2)	0.034 (2)	0.0036 (18)	0.0145 (18)	0.0059 (17)
C18A	0.040 (2)	0.048 (2)	0.043 (3)	0.0067 (19)	0.0114 (19)	0.020 (2)
C19A	0.030 (2)	0.028 (2)	0.068 (3)	−0.0009 (16)	0.012 (2)	0.013 (2)
C20A	0.027 (2)	0.0286 (19)	0.048 (2)	−0.0048 (16)	0.0033 (17)	0.0056 (17)
C21A	0.0251 (19)	0.0252 (18)	0.032 (2)	−0.0035 (15)	0.0026 (16)	0.0008 (15)
C22A	0.031 (2)	0.033 (2)	0.030 (2)	−0.0040 (16)	−0.0020 (17)	−0.0031 (16)
C23A	0.024 (2)	0.042 (2)	0.035 (2)	−0.0011 (16)	−0.0045 (17)	0.0058 (18)
C24A	0.0225 (19)	0.033 (2)	0.042 (2)	0.0056 (16)	0.0070 (17)	0.0025 (17)
C25A	0.028 (2)	0.0301 (19)	0.0258 (19)	−0.0025 (15)	0.0031 (15)	0.0013 (15)
Fe1B	0.0265 (3)	0.0251 (3)	0.0295 (3)	−0.0001 (2)	0.0060 (2)	−0.0015 (2)
Ga1B	0.0232 (2)	0.02223 (19)	0.0241 (2)	0.00140 (15)	0.00027 (16)	−0.00166 (15)
N1b	0.0228 (16)	0.0332 (16)	0.0263 (16)	0.0022 (13)	0.0009 (13)	−0.0032 (13)
Si1B	0.0365 (6)	0.0375 (6)	0.0365 (6)	0.0108 (5)	−0.0092 (5)	−0.0141 (5)
Si2B	0.0334 (6)	0.0233 (5)	0.0514 (7)	0.0015 (4)	0.0032 (5)	0.0030 (5)
Si3B	0.0233 (5)	0.0331 (5)	0.0311 (6)	0.0032 (4)	−0.0014 (4)	−0.0023 (4)
C2B	0.032 (2)	0.038 (2)	0.030 (2)	−0.0004 (17)	0.0007 (17)	0.0031 (17)
C3B	0.037 (2)	0.048 (2)	0.035 (2)	−0.0068 (19)	−0.0025 (18)	0.0110 (19)
C4B	0.039 (2)	0.068 (3)	0.026 (2)	−0.008 (2)	0.0026 (18)	0.002 (2)
C5B	0.032 (2)	0.064 (3)	0.031 (2)	−0.001 (2)	0.0026 (18)	−0.014 (2)
C6B	0.026 (2)	0.043 (2)	0.028 (2)	0.0008 (16)	0.0014 (16)	−0.0089 (17)
C7B	0.028 (2)	0.0235 (18)	0.033 (2)	0.0039 (14)	−0.0009 (16)	−0.0018 (15)
C8B	0.069 (3)	0.039 (2)	0.071 (3)	−0.002 (2)	−0.032 (3)	−0.013 (2)
C9B	0.066 (3)	0.088 (4)	0.043 (3)	0.035 (3)	−0.007 (2)	−0.033 (3)
C10B	0.037 (2)	0.026 (2)	0.104 (4)	−0.0068 (18)	0.008 (2)	0.009 (2)
C11B	0.053 (3)	0.026 (2)	0.086 (4)	0.0087 (19)	0.002 (3)	0.002 (2)
C12B	0.064 (3)	0.055 (3)	0.045 (3)	−0.008 (2)	0.013 (2)	0.015 (2)
C13B	0.029 (2)	0.051 (2)	0.044 (2)	−0.0010 (18)	−0.0049 (18)	−0.0041 (19)
C14B	0.039 (3)	0.060 (3)	0.053 (3)	0.016 (2)	−0.009 (2)	−0.007 (2)
C15B	0.031 (2)	0.061 (3)	0.048 (3)	−0.006 (2)	0.0045 (19)	0.007 (2)
C16B	0.0242 (19)	0.0241 (18)	0.039 (2)	−0.0042 (14)	0.0029 (16)	−0.0010 (15)
C17B	0.037 (2)	0.031 (2)	0.042 (2)	−0.0061 (17)	0.0096 (18)	0.0056 (17)
C18B	0.038 (2)	0.042 (2)	0.051 (3)	−0.0070 (18)	0.023 (2)	−0.004 (2)
C19B	0.024 (2)	0.033 (2)	0.057 (3)	0.0022 (16)	0.0098 (18)	−0.0003 (19)
C20B	0.0223 (19)	0.034 (2)	0.037 (2)	−0.0020 (15)	−0.0026 (16)	0.0001 (16)
C21B	0.0238 (19)	0.0278 (18)	0.032 (2)	−0.0029 (14)	0.0023 (16)	−0.0037 (15)
C22B	0.036 (2)	0.0289 (19)	0.032 (2)	0.0010 (16)	−0.0068 (17)	−0.0033 (16)
C23B	0.045 (2)	0.036 (2)	0.029 (2)	0.0000 (18)	0.0047 (18)	−0.0105 (17)
C24B	0.033 (2)	0.0245 (18)	0.042 (2)	−0.0017 (15)	0.0032 (18)	−0.0077 (16)
C25B	0.0266 (19)	0.0262 (18)	0.030 (2)	−0.0051 (15)	0.0012 (16)	−0.0036 (15)

### Geometric parameters (Å, °)

**Table:**

Fe1A—C17A	2.019 (3)	Fe1B—C22B	2.024 (4)
Fe1A—C22A	2.029 (3)	Fe1B—C17B	2.026 (3)
Fe1A—C25A	2.029 (3)	Fe1B—C25B	2.034 (3)
Fe1A—C16A	2.037 (3)	Fe1B—C20B	2.036 (4)
Fe1A—C20A	2.037 (4)	Fe1B—C21B	2.038 (3)
Fe1A—C21A	2.047 (3)	Fe1B—C16B	2.047 (3)
Fe1A—C18A	2.049 (4)	Fe1B—C18B	2.051 (4)
Fe1A—C23A	2.057 (4)	Fe1B—C19B	2.064 (4)
Fe1A—C24A	2.058 (3)	Fe1B—C24B	2.068 (3)
Fe1A—C19A	2.067 (4)	Fe1B—C23B	2.075 (4)
Fe1A—Ga1A	2.8200 (6)	Fe1B—Ga1B	2.8328 (6)
Ga1A—C16A	2.018 (3)	Ga1B—C21B	2.023 (3)
Ga1A—C21A	2.023 (3)	Ga1B—C16B	2.026 (3)
Ga1A—C7A	2.043 (3)	Ga1B—C7B	2.038 (3)
Ga1A—N1A	2.084 (3)	Ga1B—N1b	2.050 (3)
N1A—C2A	1.342 (4)	N1b—C2B	1.342 (4)
N1A—C6A	1.359 (4)	N1b—C6B	1.358 (4)
Si1A—C7A	1.862 (3)	Si1B—C7B	1.863 (4)
Si1A—C9A	1.864 (4)	Si1B—C8B	1.875 (4)
Si1A—C8A	1.871 (4)	Si1B—C9B	1.877 (4)
Si1A—C6A	1.904 (3)	Si1B—C6B	1.916 (4)
Si2A—C12A	1.869 (4)	Si2B—C10B	1.871 (4)
Si2A—C11A	1.872 (4)	Si2B—C12B	1.875 (4)
Si2A—C10A	1.873 (4)	Si2B—C11B	1.883 (4)
Si2A—C7A	1.904 (3)	Si2B—C7B	1.884 (4)
Si3A—C13A	1.875 (4)	Si3B—C13B	1.865 (4)
Si3A—C15A	1.877 (4)	Si3B—C15B	1.871 (4)
Si3A—C14A	1.884 (4)	Si3B—C14B	1.883 (4)
Si3A—C7A	1.885 (3)	Si3B—C7B	1.903 (4)
C2A—C3A	1.369 (5)	C2B—C3B	1.375 (5)
C2A—H2A	0.9500	C2B—H2B	0.9500
C3A—C4A	1.376 (5)	C3B—C4B	1.373 (5)
C3A—H3A	0.9500	C3B—H3B	0.9500
C4A—C5A	1.388 (5)	C4B—C5B	1.390 (6)
C4A—H4A	0.9500	C4B—H4B	0.9500
C5A—C6A	1.387 (5)	C5B—C6B	1.391 (5)
C5A—H5A	0.9500	C5B—H5B	0.9500
C8A—H8A	0.9800	C8B—H8D	0.9800
C8A—H8B	0.9800	C8B—H8E	0.9800
C8A—H8C	0.9800	C8B—H8F	0.9800
C9A—H9A	0.9800	C9B—H9D	0.9800
C9A—H9B	0.9800	C9B—H9E	0.9800
C9A—H9C	0.9800	C9B—H9F	0.9800
C10A—H10A	0.9800	C10B—H10D	0.9800
C10A—H10B	0.9800	C10B—H10E	0.9800
C10A—H10C	0.9800	C10B—H10F	0.9800
C11A—H11A	0.9800	C11B—H11D	0.9800
C11A—H11B	0.9800	C11B—H11E	0.9800
C11A—H11C	0.9800	C11B—H11F	0.9800
C12A—H12A	0.9800	C12B—H12D	0.9800
C12A—H12B	0.9800	C12B—H12E	0.9800
C12A—H12C	0.9800	C12B—H12F	0.9800
C13A—H13A	0.9800	C13B—H13D	0.9800
C13A—H13B	0.9800	C13B—H13E	0.9800
C13A—H13C	0.9800	C13B—H13F	0.9800
C14A—H14A	0.9800	C14B—H14D	0.9800
C14A—H14B	0.9800	C14B—H14E	0.9800
C14A—H14C	0.9800	C14B—H14F	0.9800
C15A—H15A	0.9800	C15B—H15D	0.9800
C15A—H15B	0.9800	C15B—H15E	0.9800
C15A—H15C	0.9800	C15B—H15F	0.9800
C16A—C20A	1.441 (5)	C16B—C17B	1.444 (5)
C16A—C17A	1.442 (5)	C16B—C20B	1.454 (5)
C17A—C18A	1.419 (5)	C17B—C18B	1.425 (5)
C17A—H17A	0.9500	C17B—H17B	0.9500
C18A—C19A	1.410 (6)	C18B—C19B	1.408 (5)
C18A—H18A	0.9500	C18B—H18B	0.9500
C19A—C20A	1.423 (5)	C19B—C20B	1.420 (5)
C19A—H19A	0.9500	C19B—H19B	0.9500
C20A—H20A	0.9500	C20B—H20B	0.9500
C21A—C25A	1.444 (5)	C21B—C22B	1.436 (5)
C21A—C22A	1.454 (5)	C21B—C25B	1.441 (5)
C22A—C23A	1.420 (5)	C22B—C23B	1.421 (5)
C22A—H22A	0.9500	C22B—H22B	0.9500
C23A—C24A	1.412 (5)	C23B—C24B	1.418 (5)
C23A—H23A	0.9500	C23B—H23B	0.9500
C24A—C25A	1.427 (5)	C24B—C25B	1.416 (5)
C24A—H24A	0.9500	C24B—H24B	0.9500
C25A—H25A	0.9500	C25B—H25B	0.9500
			
C17A—Fe1A—C22A	100.82 (15)	C22B—Fe1B—C17B	99.90 (15)
C17A—Fe1A—C25A	146.12 (14)	C22B—Fe1B—C25B	68.11 (14)
C22A—Fe1A—C25A	68.32 (14)	C17B—Fe1B—C25B	148.87 (14)
C17A—Fe1A—C16A	41.65 (14)	C22B—Fe1B—C20B	147.77 (14)
C22A—Fe1A—C16A	112.84 (14)	C17B—Fe1B—C20B	68.45 (15)
C25A—Fe1A—C16A	111.08 (14)	C25B—Fe1B—C20B	105.80 (14)
C17A—Fe1A—C20A	68.19 (16)	C22B—Fe1B—C21B	41.39 (14)
C22A—Fe1A—C20A	151.37 (14)	C17B—Fe1B—C21B	110.61 (14)
C25A—Fe1A—C20A	105.44 (15)	C25B—Fe1B—C21B	41.45 (13)
C16A—Fe1A—C20A	41.42 (14)	C20B—Fe1B—C21B	112.66 (14)
C17A—Fe1A—C21A	108.92 (14)	C22B—Fe1B—C16B	109.66 (14)
C22A—Fe1A—C21A	41.81 (14)	C17B—Fe1B—C16B	41.54 (14)
C25A—Fe1A—C21A	41.49 (13)	C25B—Fe1B—C16B	113.43 (13)
C16A—Fe1A—C21A	91.43 (13)	C20B—Fe1B—C16B	41.72 (13)
C20A—Fe1A—C21A	114.73 (14)	C21B—Fe1B—C16B	91.17 (13)
C17A—Fe1A—C18A	40.82 (14)	C22B—Fe1B—C18B	123.19 (16)
C22A—Fe1A—C18A	121.54 (17)	C17B—Fe1B—C18B	40.89 (14)
C25A—Fe1A—C18A	169.32 (16)	C25B—Fe1B—C18B	167.27 (15)
C16A—Fe1A—C18A	70.13 (15)	C20B—Fe1B—C18B	68.09 (16)
C20A—Fe1A—C18A	68.05 (16)	C21B—Fe1B—C18B	150.78 (15)
C21A—Fe1A—C18A	148.46 (15)	C16B—Fe1B—C18B	70.03 (14)
C17A—Fe1A—C23A	125.00 (16)	C22B—Fe1B—C19B	163.05 (15)
C22A—Fe1A—C23A	40.68 (14)	C17B—Fe1B—C19B	68.06 (15)
C25A—Fe1A—C23A	68.10 (14)	C25B—Fe1B—C19B	128.30 (15)
C16A—Fe1A—C23A	153.12 (14)	C20B—Fe1B—C19B	40.51 (14)
C20A—Fe1A—C23A	164.90 (15)	C21B—Fe1B—C19B	152.85 (15)
C21A—Fe1A—C23A	70.15 (14)	C16B—Fe1B—C19B	69.82 (14)
C18A—Fe1A—C23A	115.95 (16)	C18B—Fe1B—C19B	40.01 (15)
C17A—Fe1A—C24A	165.05 (16)	C22B—Fe1B—C24B	67.66 (14)
C22A—Fe1A—C24A	68.21 (14)	C17B—Fe1B—C24B	161.87 (15)
C25A—Fe1A—C24A	40.85 (13)	C25B—Fe1B—C24B	40.39 (13)
C16A—Fe1A—C24A	151.13 (14)	C20B—Fe1B—C24B	129.08 (15)
C20A—Fe1A—C24A	126.26 (15)	C21B—Fe1B—C24B	69.42 (13)
C21A—Fe1A—C24A	70.21 (13)	C16B—Fe1B—C24B	153.66 (14)
C18A—Fe1A—C24A	135.74 (15)	C18B—Fe1B—C24B	134.56 (15)
C23A—Fe1A—C24A	40.14 (14)	C19B—Fe1B—C24B	120.68 (15)
C17A—Fe1A—C19A	68.01 (16)	C22B—Fe1B—C23B	40.54 (13)
C22A—Fe1A—C19A	160.83 (16)	C17B—Fe1B—C23B	122.08 (16)
C25A—Fe1A—C19A	129.63 (16)	C25B—Fe1B—C23B	67.98 (14)
C16A—Fe1A—C19A	69.83 (14)	C20B—Fe1B—C23B	168.41 (14)
C20A—Fe1A—C19A	40.58 (15)	C21B—Fe1B—C23B	69.69 (14)
C21A—Fe1A—C19A	155.22 (15)	C16B—Fe1B—C23B	149.26 (14)
C18A—Fe1A—C19A	40.06 (16)	C18B—Fe1B—C23B	115.85 (16)
C23A—Fe1A—C19A	132.79 (15)	C19B—Fe1B—C23B	135.39 (15)
C24A—Fe1A—C19A	119.31 (15)	C24B—Fe1B—C23B	40.03 (14)
C17A—Fe1A—Ga1A	72.79 (10)	C22B—Fe1B—Ga1B	73.20 (10)
C22A—Fe1A—Ga1A	75.67 (10)	C17B—Fe1B—Ga1B	74.00 (10)
C25A—Fe1A—Ga1A	73.41 (10)	C25B—Fe1B—Ga1B	75.00 (9)
C16A—Fe1A—Ga1A	45.66 (9)	C20B—Fe1B—Ga1B	74.67 (10)
C20A—Fe1A—Ga1A	75.81 (10)	C21B—Fe1B—Ga1B	45.55 (9)
C21A—Fe1A—Ga1A	45.78 (10)	C16B—Fe1B—Ga1B	45.64 (10)
C18A—Fe1A—Ga1A	111.81 (11)	C18B—Fe1B—Ga1B	112.63 (11)
C23A—Fe1A—Ga1A	113.63 (10)	C19B—Fe1B—Ga1B	112.70 (11)
C24A—Fe1A—Ga1A	112.37 (10)	C24B—Fe1B—Ga1B	112.63 (10)
C19A—Fe1A—Ga1A	113.50 (11)	C23B—Fe1B—Ga1B	111.74 (10)
C16A—Ga1A—C21A	92.68 (13)	C21B—Ga1B—C16B	92.22 (13)
C16A—Ga1A—C7A	126.52 (13)	C21B—Ga1B—C7B	123.85 (14)
C21A—Ga1A—C7A	127.08 (13)	C16B—Ga1B—C7B	128.70 (13)
C16A—Ga1A—N1A	108.52 (13)	C21B—Ga1B—N1b	109.34 (12)
C21A—Ga1A—N1A	104.33 (12)	C16B—Ga1B—N1b	103.82 (13)
C7A—Ga1A—N1A	95.63 (12)	C7B—Ga1B—N1b	97.21 (12)
C16A—Ga1A—Fe1A	46.20 (10)	C21B—Ga1B—Fe1B	45.99 (9)
C21A—Ga1A—Fe1A	46.49 (9)	C16B—Ga1B—Fe1B	46.24 (9)
C7A—Ga1A—Fe1A	150.96 (10)	C7B—Ga1B—Fe1B	149.22 (10)
N1A—Ga1A—Fe1A	113.39 (7)	N1b—Ga1B—Fe1B	113.56 (8)
C2A—N1A—C6A	119.4 (3)	C2B—N1b—C6B	119.9 (3)
C2A—N1A—Ga1A	127.2 (2)	C2B—N1b—Ga1B	125.4 (2)
C6A—N1A—Ga1A	113.4 (2)	C6B—N1b—Ga1B	114.7 (2)
C7A—Si1A—C9A	115.99 (18)	C7B—Si1B—C8B	115.92 (19)
C7A—Si1A—C8A	118.61 (18)	C7B—Si1B—C9B	119.06 (18)
C9A—Si1A—C8A	106.4 (2)	C8B—Si1B—C9B	104.3 (2)
C7A—Si1A—C6A	104.80 (15)	C7B—Si1B—C6B	105.00 (15)
C9A—Si1A—C6A	102.99 (17)	C8B—Si1B—C6B	103.99 (18)
C8A—Si1A—C6A	106.55 (17)	C9B—Si1B—C6B	107.4 (2)
C12A—Si2A—C11A	105.13 (19)	C10B—Si2B—C12B	106.0 (2)
C12A—Si2A—C10A	105.0 (2)	C10B—Si2B—C11B	106.32 (19)
C11A—Si2A—C10A	107.7 (2)	C12B—Si2B—C11B	105.7 (2)
C12A—Si2A—C7A	114.47 (17)	C10B—Si2B—C7B	113.31 (18)
C11A—Si2A—C7A	113.26 (17)	C12B—Si2B—C7B	112.28 (17)
C10A—Si2A—C7A	110.72 (17)	C11B—Si2B—C7B	112.59 (18)
C13A—Si3A—C15A	105.8 (2)	C13B—Si3B—C15B	104.13 (19)
C13A—Si3A—C14A	105.35 (19)	C13B—Si3B—C14B	104.19 (18)
C15A—Si3A—C14A	105.70 (19)	C15B—Si3B—C14B	108.8 (2)
C13A—Si3A—C7A	113.14 (17)	C13B—Si3B—C7B	116.39 (16)
C15A—Si3A—C7A	114.06 (16)	C15B—Si3B—C7B	109.75 (17)
C14A—Si3A—C7A	112.10 (17)	C14B—Si3B—C7B	112.94 (17)
N1A—C2A—C3A	122.7 (3)	N1b—C2B—C3B	122.5 (4)
N1A—C2A—H2A	118.6	N1b—C2B—H2B	118.7
C3A—C2A—H2A	118.6	C3B—C2B—H2B	118.7
C2A—C3A—C4A	119.4 (3)	C4B—C3B—C2B	118.7 (4)
C2A—C3A—H3A	120.3	C4B—C3B—H3B	120.6
C4A—C3A—H3A	120.3	C2B—C3B—H3B	120.6
C3A—C4A—C5A	118.1 (3)	C3B—C4B—C5B	119.2 (4)
C3A—C4A—H4A	121.0	C3B—C4B—H4B	120.4
C5A—C4A—H4A	121.0	C5B—C4B—H4B	120.4
C6A—C5A—C4A	120.9 (3)	C4B—C5B—C6B	120.1 (4)
C6A—C5A—H5A	119.5	C4B—C5B—H5B	119.9
C4A—C5A—H5A	119.5	C6B—C5B—H5B	119.9
N1A—C6A—C5A	119.5 (3)	N1b—C6B—C5B	119.5 (3)
N1A—C6A—Si1A	115.2 (2)	N1b—C6B—Si1B	115.3 (3)
C5A—C6A—Si1A	125.2 (3)	C5B—C6B—Si1B	125.0 (3)
Si1A—C7A—Si3A	111.64 (17)	Si1B—C7B—Si2B	112.83 (18)
Si1A—C7A—Si2A	111.30 (18)	Si1B—C7B—Si3B	110.04 (18)
Si3A—C7A—Si2A	112.52 (17)	Si2B—C7B—Si3B	114.48 (18)
Si1A—C7A—Ga1A	100.45 (15)	Si1B—C7B—Ga1B	102.21 (16)
Si3A—C7A—Ga1A	114.46 (17)	Si2B—C7B—Ga1B	110.78 (17)
Si2A—C7A—Ga1A	105.75 (15)	Si3B—C7B—Ga1B	105.57 (15)
Si1A—C8A—H8A	109.5	Si1B—C8B—H8D	109.5
Si1A—C8A—H8B	109.5	Si1B—C8B—H8E	109.5
H8A—C8A—H8B	109.5	H8D—C8B—H8E	109.5
Si1A—C8A—H8C	109.5	Si1B—C8B—H8F	109.5
H8A—C8A—H8C	109.5	H8D—C8B—H8F	109.5
H8B—C8A—H8C	109.5	H8E—C8B—H8F	109.5
Si1A—C9A—H9A	109.5	Si1B—C9B—H9D	109.5
Si1A—C9A—H9B	109.5	Si1B—C9B—H9E	109.5
H9A—C9A—H9B	109.5	H9D—C9B—H9E	109.5
Si1A—C9A—H9C	109.5	Si1B—C9B—H9F	109.5
H9A—C9A—H9C	109.5	H9D—C9B—H9F	109.5
H9B—C9A—H9C	109.5	H9E—C9B—H9F	109.5
Si2A—C10A—H10A	109.5	Si2B—C10B—H10D	109.5
Si2A—C10A—H10B	109.5	Si2B—C10B—H10E	109.5
H10A—C10A—H10B	109.5	H10D—C10B—H10E	109.5
Si2A—C10A—H10C	109.5	Si2B—C10B—H10F	109.5
H10A—C10A—H10C	109.5	H10D—C10B—H10F	109.5
H10B—C10A—H10C	109.5	H10E—C10B—H10F	109.5
Si2A—C11A—H11A	109.5	Si2B—C11B—H11D	109.5
Si2A—C11A—H11B	109.5	Si2B—C11B—H11E	109.5
H11A—C11A—H11B	109.5	H11D—C11B—H11E	109.5
Si2A—C11A—H11C	109.5	Si2B—C11B—H11F	109.5
H11A—C11A—H11C	109.5	H11D—C11B—H11F	109.5
H11B—C11A—H11C	109.5	H11E—C11B—H11F	109.5
Si2A—C12A—H12A	109.5	Si2B—C12B—H12D	109.5
Si2A—C12A—H12B	109.5	Si2B—C12B—H12E	109.5
H12A—C12A—H12B	109.5	H12D—C12B—H12E	109.5
Si2A—C12A—H12C	109.5	Si2B—C12B—H12F	109.5
H12A—C12A—H12C	109.5	H12D—C12B—H12F	109.5
H12B—C12A—H12C	109.5	H12E—C12B—H12F	109.5
Si3A—C13A—H13A	109.5	Si3B—C13B—H13D	109.5
Si3A—C13A—H13B	109.5	Si3B—C13B—H13E	109.5
H13A—C13A—H13B	109.5	H13D—C13B—H13E	109.5
Si3A—C13A—H13C	109.5	Si3B—C13B—H13F	109.5
H13A—C13A—H13C	109.5	H13D—C13B—H13F	109.5
H13B—C13A—H13C	109.5	H13E—C13B—H13F	109.5
Si3A—C14A—H14A	109.5	Si3B—C14B—H14D	109.5
Si3A—C14A—H14B	109.5	Si3B—C14B—H14E	109.5
H14A—C14A—H14B	109.5	H14D—C14B—H14E	109.5
Si3A—C14A—H14C	109.5	Si3B—C14B—H14F	109.5
H14A—C14A—H14C	109.5	H14D—C14B—H14F	109.5
H14B—C14A—H14C	109.5	H14E—C14B—H14F	109.5
Si3A—C15A—H15A	109.5	Si3B—C15B—H15D	109.5
Si3A—C15A—H15B	109.5	Si3B—C15B—H15E	109.5
H15A—C15A—H15B	109.5	H15D—C15B—H15E	109.5
Si3A—C15A—H15C	109.5	Si3B—C15B—H15F	109.5
H15A—C15A—H15C	109.5	H15D—C15B—H15F	109.5
H15B—C15A—H15C	109.5	H15E—C15B—H15F	109.5
C20A—C16A—C17A	104.1 (3)	C17B—C16B—C20B	104.1 (3)
C20A—C16A—Ga1A	122.7 (3)	C17B—C16B—Ga1B	118.3 (3)
C17A—C16A—Ga1A	115.5 (3)	C20B—C16B—Ga1B	119.5 (2)
C20A—C16A—Fe1A	69.30 (19)	C17B—C16B—Fe1B	68.46 (19)
C17A—C16A—Fe1A	68.5 (2)	C20B—C16B—Fe1B	68.75 (19)
Ga1A—C16A—Fe1A	88.14 (13)	Ga1B—C16B—Fe1B	88.12 (13)
C18A—C17A—C16A	110.2 (3)	C18B—C17B—C16B	110.1 (3)
C18A—C17A—Fe1A	70.7 (2)	C18B—C17B—Fe1B	70.5 (2)
C16A—C17A—Fe1A	69.83 (19)	C16B—C17B—Fe1B	70.00 (19)
C18A—C17A—H17A	124.9	C18B—C17B—H17B	124.9
C16A—C17A—H17A	124.9	C16B—C17B—H17B	124.9
Fe1A—C17A—H17A	126.2	Fe1B—C17B—H17B	126.1
C19A—C18A—C17A	107.8 (4)	C19B—C18B—C17B	107.8 (3)
C19A—C18A—Fe1A	70.7 (2)	C19B—C18B—Fe1B	70.5 (2)
C17A—C18A—Fe1A	68.5 (2)	C17B—C18B—Fe1B	68.6 (2)
C19A—C18A—H18A	126.1	C19B—C18B—H18B	126.1
C17A—C18A—H18A	126.1	C17B—C18B—H18B	126.1
Fe1A—C18A—H18A	126.3	Fe1B—C18B—H18B	126.4
C18A—C19A—C20A	107.6 (3)	C18B—C19B—C20B	108.1 (3)
C18A—C19A—Fe1A	69.3 (2)	C18B—C19B—Fe1B	69.5 (2)
C20A—C19A—Fe1A	68.6 (2)	C20B—C19B—Fe1B	68.7 (2)
C18A—C19A—H19A	126.2	C18B—C19B—H19B	126.0
C20A—C19A—H19A	126.2	C20B—C19B—H19B	126.0
Fe1A—C19A—H19A	127.5	Fe1B—C19B—H19B	127.4
C19A—C20A—C16A	110.2 (4)	C19B—C20B—C16B	109.9 (3)
C19A—C20A—Fe1A	70.8 (2)	C19B—C20B—Fe1B	70.8 (2)
C16A—C20A—Fe1A	69.3 (2)	C16B—C20B—Fe1B	69.5 (2)
C19A—C20A—H20A	124.9	C19B—C20B—H20B	125.0
C16A—C20A—H20A	124.9	C16B—C20B—H20B	125.0
Fe1A—C20A—H20A	126.6	Fe1B—C20B—H20B	126.2
C25A—C21A—C22A	103.7 (3)	C22B—C21B—C25B	104.4 (3)
C25A—C21A—Ga1A	116.7 (2)	C22B—C21B—Ga1B	117.2 (2)
C22A—C21A—Ga1A	121.0 (2)	C25B—C21B—Ga1B	121.1 (2)
C25A—C21A—Fe1A	68.61 (18)	C22B—C21B—Fe1B	68.79 (19)
C22A—C21A—Fe1A	68.43 (19)	C25B—C21B—Fe1B	69.13 (19)
Ga1A—C21A—Fe1A	87.73 (13)	Ga1B—C21B—Fe1B	88.46 (13)
C23A—C22A—C21A	110.3 (3)	C23B—C22B—C21B	110.7 (3)
C23A—C22A—Fe1A	70.7 (2)	C23B—C22B—Fe1B	71.7 (2)
C21A—C22A—Fe1A	69.76 (19)	C21B—C22B—Fe1B	69.8 (2)
C23A—C22A—H22A	124.9	C23B—C22B—H22B	124.6
C21A—C22A—H22A	124.9	C21B—C22B—H22B	124.6
Fe1A—C22A—H22A	126.2	Fe1B—C22B—H22B	125.5
C24A—C23A—C22A	108.0 (3)	C24B—C23B—C22B	106.8 (3)
C24A—C23A—Fe1A	70.0 (2)	C24B—C23B—Fe1B	69.7 (2)
C22A—C23A—Fe1A	68.6 (2)	C22B—C23B—Fe1B	67.8 (2)
C24A—C23A—H23A	126.0	C24B—C23B—H23B	126.6
C22A—C23A—H23A	126.0	C22B—C23B—H23B	126.6
Fe1A—C23A—H23A	127.0	Fe1B—C23B—H23B	127.4
C23A—C24A—C25A	107.4 (3)	C25B—C24B—C23B	108.3 (3)
C23A—C24A—Fe1A	69.9 (2)	C25B—C24B—Fe1B	68.52 (19)
C25A—C24A—Fe1A	68.48 (19)	C23B—C24B—Fe1B	70.3 (2)
C23A—C24A—H24A	126.3	C25B—C24B—H24B	125.8
C25A—C24A—H24A	126.3	C23B—C24B—H24B	125.8
Fe1A—C24A—H24A	126.9	Fe1B—C24B—H24B	126.9
C24A—C25A—C21A	110.6 (3)	C24B—C25B—C21B	109.8 (3)
C24A—C25A—Fe1A	70.67 (19)	C24B—C25B—Fe1B	71.1 (2)
C21A—C25A—Fe1A	69.90 (19)	C21B—C25B—Fe1B	69.42 (19)
C24A—C25A—H25A	124.7	C24B—C25B—H25B	125.1
C21A—C25A—H25A	124.7	C21B—C25B—H25B	125.1
Fe1A—C25A—H25A	126.4	Fe1B—C25B—H25B	126.0
			
C17A—Fe1A—Ga1A—C16A	−37.72 (18)	C22B—Fe1B—Ga1B—C21B	36.71 (17)
C22A—Fe1A—Ga1A—C16A	−144.10 (18)	C17B—Fe1B—Ga1B—C21B	142.58 (18)
C25A—Fe1A—Ga1A—C16A	144.67 (18)	C25B—Fe1B—Ga1B—C21B	−34.49 (17)
C20A—Fe1A—Ga1A—C16A	33.45 (18)	C20B—Fe1B—Ga1B—C21B	−145.95 (17)
C21A—Fe1A—Ga1A—C16A	−178.5 (2)	C16B—Fe1B—Ga1B—C21B	178.6 (2)
C18A—Fe1A—Ga1A—C16A	−25.5 (2)	C18B—Fe1B—Ga1B—C21B	156.29 (19)
C23A—Fe1A—Ga1A—C16A	−159.04 (18)	C19B—Fe1B—Ga1B—C21B	−160.19 (18)
C24A—Fe1A—Ga1A—C16A	157.20 (18)	C24B—Fe1B—Ga1B—C21B	−19.52 (18)
C19A—Fe1A—Ga1A—C16A	18.08 (19)	C23B—Fe1B—Ga1B—C21B	23.85 (18)
C17A—Fe1A—Ga1A—C21A	140.81 (18)	C22B—Fe1B—Ga1B—C16B	−141.88 (18)
C22A—Fe1A—Ga1A—C21A	34.43 (17)	C17B—Fe1B—Ga1B—C16B	−36.02 (18)
C25A—Fe1A—Ga1A—C21A	−36.80 (17)	C25B—Fe1B—Ga1B—C16B	146.91 (17)
C16A—Fe1A—Ga1A—C21A	178.5 (2)	C20B—Fe1B—Ga1B—C16B	35.46 (17)
C20A—Fe1A—Ga1A—C21A	−148.02 (17)	C21B—Fe1B—Ga1B—C16B	−178.6 (2)
C18A—Fe1A—Ga1A—C21A	153.07 (19)	C18B—Fe1B—Ga1B—C16B	−22.30 (19)
C23A—Fe1A—Ga1A—C21A	19.48 (17)	C19B—Fe1B—Ga1B—C16B	21.22 (18)
C24A—Fe1A—Ga1A—C21A	−24.27 (18)	C24B—Fe1B—Ga1B—C16B	161.89 (18)
C19A—Fe1A—Ga1A—C21A	−163.40 (19)	C23B—Fe1B—Ga1B—C16B	−154.74 (18)
C17A—Fe1A—Ga1A—C7A	50.7 (2)	C22B—Fe1B—Ga1B—C7B	−47.1 (2)
C22A—Fe1A—Ga1A—C7A	−55.7 (2)	C17B—Fe1B—Ga1B—C7B	58.8 (2)
C25A—Fe1A—Ga1A—C7A	−127.0 (2)	C25B—Fe1B—Ga1B—C7B	−118.3 (2)
C16A—Fe1A—Ga1A—C7A	88.4 (2)	C20B—Fe1B—Ga1B—C7B	130.3 (2)
C20A—Fe1A—Ga1A—C7A	121.8 (2)	C21B—Fe1B—Ga1B—C7B	−83.8 (2)
C21A—Fe1A—Ga1A—C7A	−90.2 (2)	C16B—Fe1B—Ga1B—C7B	94.8 (2)
C18A—Fe1A—Ga1A—C7A	62.9 (2)	C18B—Fe1B—Ga1B—C7B	72.5 (2)
C23A—Fe1A—Ga1A—C7A	−70.7 (2)	C19B—Fe1B—Ga1B—C7B	116.0 (2)
C24A—Fe1A—Ga1A—C7A	−114.4 (2)	C24B—Fe1B—Ga1B—C7B	−103.3 (2)
C19A—Fe1A—Ga1A—C7A	106.4 (2)	C23B—Fe1B—Ga1B—C7B	−59.9 (2)
C17A—Fe1A—Ga1A—N1A	−131.42 (15)	C22B—Fe1B—Ga1B—N1b	131.36 (14)
C22A—Fe1A—Ga1A—N1A	122.20 (13)	C17B—Fe1B—Ga1B—N1b	−122.77 (15)
C25A—Fe1A—Ga1A—N1A	50.97 (13)	C25B—Fe1B—Ga1B—N1b	60.16 (14)
C16A—Fe1A—Ga1A—N1A	−93.71 (16)	C20B—Fe1B—Ga1B—N1b	−51.29 (14)
C20A—Fe1A—Ga1A—N1A	−60.25 (14)	C21B—Fe1B—Ga1B—N1b	94.65 (16)
C21A—Fe1A—Ga1A—N1A	87.77 (16)	C16B—Fe1B—Ga1B—N1b	−86.75 (16)
C18A—Fe1A—Ga1A—N1A	−119.16 (16)	C18B—Fe1B—Ga1B—N1b	−109.05 (16)
C23A—Fe1A—Ga1A—N1A	107.26 (14)	C19B—Fe1B—Ga1B—N1b	−65.53 (14)
C24A—Fe1A—Ga1A—N1A	63.50 (14)	C24B—Fe1B—Ga1B—N1b	75.14 (14)
C19A—Fe1A—Ga1A—N1A	−75.63 (16)	C23B—Fe1B—Ga1B—N1b	118.51 (14)
C16A—Ga1A—N1A—C2A	−31.2 (3)	C21B—Ga1B—N1b—C2B	39.6 (3)
C21A—Ga1A—N1A—C2A	66.7 (3)	C16B—Ga1B—N1b—C2B	−57.8 (3)
C7A—Ga1A—N1A—C2A	−162.7 (3)	C7B—Ga1B—N1b—C2B	169.3 (3)
Fe1A—Ga1A—N1A—C2A	18.3 (3)	Fe1B—Ga1B—N1b—C2B	−9.8 (3)
C16A—Ga1A—N1A—C6A	151.7 (2)	C21B—Ga1B—N1b—C6B	−142.0 (2)
C21A—Ga1A—N1A—C6A	−110.5 (2)	C16B—Ga1B—N1b—C6B	120.6 (2)
C7A—Ga1A—N1A—C6A	20.1 (2)	C7B—Ga1B—N1b—C6B	−12.3 (3)
Fe1A—Ga1A—N1A—C6A	−158.9 (2)	Fe1B—Ga1B—N1b—C6B	168.5 (2)
C6A—N1A—C2A—C3A	−0.5 (5)	C6B—N1b—C2B—C3B	−1.0 (5)
Ga1A—N1A—C2A—C3A	−177.5 (3)	Ga1B—N1b—C2B—C3B	177.3 (3)
N1A—C2A—C3A—C4A	0.9 (6)	N1b—C2B—C3B—C4B	0.6 (6)
C2A—C3A—C4A—C5A	−0.4 (6)	C2B—C3B—C4B—C5B	0.3 (6)
C3A—C4A—C5A—C6A	−0.4 (6)	C3B—C4B—C5B—C6B	−0.8 (6)
C2A—N1A—C6A—C5A	−0.4 (5)	C2B—N1b—C6B—C5B	0.5 (5)
Ga1A—N1A—C6A—C5A	177.0 (3)	Ga1B—N1b—C6B—C5B	−177.9 (3)
C2A—N1A—C6A—Si1A	−178.9 (2)	C2B—N1b—C6B—Si1B	176.4 (3)
Ga1A—N1A—C6A—Si1A	−1.5 (3)	Ga1B—N1b—C6B—Si1B	−2.0 (3)
C4A—C5A—C6A—N1A	0.8 (5)	C4B—C5B—C6B—N1b	0.4 (6)
C4A—C5A—C6A—Si1A	179.1 (3)	C4B—C5B—C6B—Si1B	−175.1 (3)
C7A—Si1A—C6A—N1A	−20.3 (3)	C7B—Si1B—C6B—N1b	17.3 (3)
C9A—Si1A—C6A—N1A	101.4 (3)	C8B—Si1B—C6B—N1b	−104.9 (3)
C8A—Si1A—C6A—N1A	−146.9 (3)	C9B—Si1B—C6B—N1b	144.9 (3)
C7A—Si1A—C6A—C5A	161.3 (3)	C7B—Si1B—C6B—C5B	−167.1 (3)
C9A—Si1A—C6A—C5A	−77.0 (3)	C8B—Si1B—C6B—C5B	70.7 (4)
C8A—Si1A—C6A—C5A	34.7 (4)	C9B—Si1B—C6B—C5B	−39.5 (4)
C9A—Si1A—C7A—Si3A	39.3 (2)	C8B—Si1B—C7B—Si2B	−27.6 (3)
C8A—Si1A—C7A—Si3A	−89.2 (2)	C9B—Si1B—C7B—Si2B	98.2 (3)
C6A—Si1A—C7A—Si3A	152.12 (17)	C6B—Si1B—C7B—Si2B	−141.67 (18)
C9A—Si1A—C7A—Si2A	165.97 (19)	C8B—Si1B—C7B—Si3B	−156.77 (18)
C8A—Si1A—C7A—Si2A	37.4 (3)	C9B—Si1B—C7B—Si3B	−31.0 (3)
C6A—Si1A—C7A—Si2A	−81.23 (18)	C6B—Si1B—C7B—Si3B	89.12 (19)
C9A—Si1A—C7A—Ga1A	−82.4 (2)	C8B—Si1B—C7B—Ga1B	91.4 (2)
C8A—Si1A—C7A—Ga1A	149.05 (18)	C9B—Si1B—C7B—Ga1B	−142.8 (2)
C6A—Si1A—C7A—Ga1A	30.37 (18)	C6B—Si1B—C7B—Ga1B	−22.67 (19)
C13A—Si3A—C7A—Si1A	171.91 (19)	C10B—Si2B—C7B—Si1B	65.3 (2)
C15A—Si3A—C7A—Si1A	−67.1 (2)	C12B—Si2B—C7B—Si1B	−174.6 (2)
C14A—Si3A—C7A—Si1A	52.9 (2)	C11B—Si2B—C7B—Si1B	−55.4 (3)
C13A—Si3A—C7A—Si2A	45.9 (2)	C10B—Si2B—C7B—Si3B	−167.81 (19)
C15A—Si3A—C7A—Si2A	166.88 (18)	C12B—Si2B—C7B—Si3B	−47.7 (2)
C14A—Si3A—C7A—Si2A	−73.0 (2)	C11B—Si2B—C7B—Si3B	71.5 (2)
C13A—Si3A—C7A—Ga1A	−74.8 (2)	C10B—Si2B—C7B—Ga1B	−48.6 (2)
C15A—Si3A—C7A—Ga1A	46.1 (2)	C12B—Si2B—C7B—Ga1B	71.5 (2)
C14A—Si3A—C7A—Ga1A	166.20 (18)	C11B—Si2B—C7B—Ga1B	−169.30 (19)
C12A—Si2A—C7A—Si1A	164.59 (18)	C13B—Si3B—C7B—Si1B	−169.17 (17)
C11A—Si2A—C7A—Si1A	−74.9 (2)	C15B—Si3B—C7B—Si1B	−51.3 (2)
C10A—Si2A—C7A—Si1A	46.2 (2)	C14B—Si3B—C7B—Si1B	70.3 (2)
C12A—Si2A—C7A—Si3A	−69.2 (2)	C13B—Si3B—C7B—Si2B	62.5 (2)
C11A—Si2A—C7A—Si3A	51.3 (2)	C15B—Si3B—C7B—Si2B	−179.59 (19)
C10A—Si2A—C7A—Si3A	172.32 (19)	C14B—Si3B—C7B—Si2B	−58.0 (2)
C12A—Si2A—C7A—Ga1A	56.4 (2)	C13B—Si3B—C7B—Ga1B	−59.6 (2)
C11A—Si2A—C7A—Ga1A	176.91 (18)	C15B—Si3B—C7B—Ga1B	58.3 (2)
C10A—Si2A—C7A—Ga1A	−62.0 (2)	C14B—Si3B—C7B—Ga1B	179.93 (17)
C16A—Ga1A—C7A—Si1A	−146.69 (15)	C21B—Ga1B—C7B—Si1B	139.57 (15)
C21A—Ga1A—C7A—Si1A	84.1 (2)	C16B—Ga1B—C7B—Si1B	−93.8 (2)
N1A—Ga1A—C7A—Si1A	−28.65 (15)	N1b—Ga1B—C7B—Si1B	20.43 (16)
Fe1A—Ga1A—C7A—Si1A	149.43 (10)	Fe1B—Ga1B—C7B—Si1B	−161.02 (8)
C16A—Ga1A—C7A—Si3A	93.6 (2)	C21B—Ga1B—C7B—Si2B	−99.99 (19)
C21A—Ga1A—C7A—Si3A	−35.7 (2)	C16B—Ga1B—C7B—Si2B	26.7 (3)
N1A—Ga1A—C7A—Si3A	−148.37 (16)	N1b—Ga1B—C7B—Si2B	140.88 (16)
Fe1A—Ga1A—C7A—Si3A	29.7 (3)	Fe1B—Ga1B—C7B—Si2B	−40.6 (3)
C16A—Ga1A—C7A—Si2A	−30.8 (2)	C21B—Ga1B—C7B—Si3B	24.5 (2)
C21A—Ga1A—C7A—Si2A	−160.09 (14)	C16B—Ga1B—C7B—Si3B	151.13 (15)
N1A—Ga1A—C7A—Si2A	87.19 (16)	N1b—Ga1B—C7B—Si3B	−94.67 (16)
Fe1A—Ga1A—C7A—Si2A	−94.7 (2)	Fe1B—Ga1B—C7B—Si3B	83.9 (2)
C21A—Ga1A—C16A—C20A	−64.9 (3)	C21B—Ga1B—C16B—C17B	63.2 (3)
C7A—Ga1A—C16A—C20A	153.3 (3)	C7B—Ga1B—C16B—C17B	−75.0 (3)
N1A—Ga1A—C16A—C20A	41.2 (3)	N1b—Ga1B—C16B—C17B	173.8 (2)
Fe1A—Ga1A—C16A—C20A	−63.8 (3)	Fe1B—Ga1B—C16B—C17B	64.2 (2)
C21A—Ga1A—C16A—C17A	64.0 (3)	C21B—Ga1B—C16B—C20B	−65.2 (3)
C7A—Ga1A—C16A—C17A	−77.8 (3)	C7B—Ga1B—C16B—C20B	156.6 (2)
N1A—Ga1A—C16A—C17A	170.1 (2)	N1b—Ga1B—C16B—C20B	45.3 (3)
Fe1A—Ga1A—C16A—C17A	65.1 (2)	Fe1B—Ga1B—C16B—C20B	−64.2 (2)
C21A—Ga1A—C16A—Fe1A	−1.07 (14)	C21B—Ga1B—C16B—Fe1B	−1.01 (14)
C7A—Ga1A—C16A—Fe1A	−142.86 (13)	C7B—Ga1B—C16B—Fe1B	−139.20 (14)
N1A—Ga1A—C16A—Fe1A	105.01 (13)	N1b—Ga1B—C16B—Fe1B	109.53 (12)
C17A—Fe1A—C16A—C20A	−115.4 (3)	C22B—Fe1B—C16B—C17B	−82.7 (2)
C22A—Fe1A—C16A—C20A	164.2 (2)	C25B—Fe1B—C16B—C17B	−156.6 (2)
C25A—Fe1A—C16A—C20A	89.7 (2)	C20B—Fe1B—C16B—C17B	115.7 (3)
C21A—Fe1A—C16A—C20A	127.2 (2)	C21B—Fe1B—C16B—C17B	−120.5 (2)
C18A—Fe1A—C16A—C20A	−79.0 (2)	C18B—Fe1B—C16B—C17B	36.6 (2)
C23A—Fe1A—C16A—C20A	172.6 (3)	C19B—Fe1B—C16B—C17B	79.3 (2)
C24A—Fe1A—C16A—C20A	78.2 (3)	C24B—Fe1B—C16B—C17B	−161.8 (3)
C19A—Fe1A—C16A—C20A	−36.2 (2)	C23B—Fe1B—C16B—C17B	−70.7 (3)
Ga1A—Fe1A—C16A—C20A	126.1 (2)	Ga1B—Fe1B—C16B—C17B	−121.5 (2)
C22A—Fe1A—C16A—C17A	−80.4 (2)	C22B—Fe1B—C16B—C20B	161.6 (2)
C25A—Fe1A—C16A—C17A	−154.9 (2)	C17B—Fe1B—C16B—C20B	−115.7 (3)
C20A—Fe1A—C16A—C17A	115.4 (3)	C25B—Fe1B—C16B—C20B	87.7 (2)
C21A—Fe1A—C16A—C17A	−117.4 (2)	C21B—Fe1B—C16B—C20B	123.8 (2)
C18A—Fe1A—C16A—C17A	36.5 (2)	C18B—Fe1B—C16B—C20B	−79.1 (2)
C23A—Fe1A—C16A—C17A	−72.0 (4)	C19B—Fe1B—C16B—C20B	−36.4 (2)
C24A—Fe1A—C16A—C17A	−166.4 (3)	C24B—Fe1B—C16B—C20B	82.5 (4)
C19A—Fe1A—C16A—C17A	79.2 (2)	C23B—Fe1B—C16B—C20B	173.6 (3)
Ga1A—Fe1A—C16A—C17A	−118.4 (2)	Ga1B—Fe1B—C16B—C20B	122.8 (2)
C17A—Fe1A—C16A—Ga1A	118.4 (2)	C22B—Fe1B—C16B—Ga1B	38.86 (17)
C22A—Fe1A—C16A—Ga1A	38.06 (18)	C17B—Fe1B—C16B—Ga1B	121.5 (2)
C25A—Fe1A—C16A—Ga1A	−36.44 (17)	C25B—Fe1B—C16B—Ga1B	−35.08 (17)
C20A—Fe1A—C16A—Ga1A	−126.1 (3)	C20B—Fe1B—C16B—Ga1B	−122.8 (2)
C21A—Fe1A—C16A—Ga1A	1.06 (14)	C21B—Fe1B—C16B—Ga1B	1.00 (14)
C18A—Fe1A—C16A—Ga1A	154.89 (19)	C18B—Fe1B—C16B—Ga1B	158.12 (18)
C23A—Fe1A—C16A—Ga1A	46.5 (4)	C19B—Fe1B—C16B—Ga1B	−159.16 (17)
C24A—Fe1A—C16A—Ga1A	−47.9 (3)	C24B—Fe1B—C16B—Ga1B	−40.3 (4)
C19A—Fe1A—C16A—Ga1A	−162.35 (19)	C23B—Fe1B—C16B—Ga1B	50.9 (3)
C20A—C16A—C17A—C18A	1.3 (4)	C20B—C16B—C17B—C18B	0.7 (4)
Ga1A—C16A—C17A—C18A	−136.2 (3)	Ga1B—C16B—C17B—C18B	−134.6 (3)
Fe1A—C16A—C17A—C18A	−59.3 (3)	Fe1B—C16B—C17B—C18B	−59.2 (3)
C20A—C16A—C17A—Fe1A	60.6 (2)	C20B—C16B—C17B—Fe1B	60.0 (2)
Ga1A—C16A—C17A—Fe1A	−76.90 (19)	Ga1B—C16B—C17B—Fe1B	−75.4 (2)
C22A—Fe1A—C17A—C18A	−126.4 (3)	C22B—Fe1B—C17B—C18B	−130.3 (2)
C25A—Fe1A—C17A—C18A	166.6 (3)	C25B—Fe1B—C17B—C18B	166.0 (3)
C16A—Fe1A—C17A—C18A	121.3 (3)	C20B—Fe1B—C17B—C18B	81.0 (3)
C20A—Fe1A—C17A—C18A	81.2 (3)	C21B—Fe1B—C17B—C18B	−171.9 (2)
C21A—Fe1A—C17A—C18A	−169.0 (2)	C16B—Fe1B—C17B—C18B	121.1 (3)
C23A—Fe1A—C17A—C18A	−90.4 (3)	C19B—Fe1B—C17B—C18B	37.2 (2)
C24A—Fe1A—C17A—C18A	−84.9 (6)	C24B—Fe1B—C17B—C18B	−85.3 (5)
C19A—Fe1A—C17A—C18A	37.3 (2)	C23B—Fe1B—C17B—C18B	−93.6 (3)
Ga1A—Fe1A—C17A—C18A	162.4 (3)	Ga1B—Fe1B—C17B—C18B	160.5 (2)
C22A—Fe1A—C17A—C16A	112.3 (2)	C22B—Fe1B—C17B—C16B	108.5 (2)
C25A—Fe1A—C17A—C16A	45.3 (4)	C25B—Fe1B—C17B—C16B	44.8 (4)
C20A—Fe1A—C17A—C16A	−40.1 (2)	C20B—Fe1B—C17B—C16B	−40.1 (2)
C21A—Fe1A—C17A—C16A	69.8 (2)	C21B—Fe1B—C17B—C16B	67.0 (2)
C18A—Fe1A—C17A—C16A	−121.3 (3)	C18B—Fe1B—C17B—C16B	−121.1 (3)
C23A—Fe1A—C17A—C16A	148.3 (2)	C19B—Fe1B—C17B—C16B	−83.9 (2)
C24A—Fe1A—C17A—C16A	153.8 (5)	C24B—Fe1B—C17B—C16B	153.6 (4)
C19A—Fe1A—C17A—C16A	−83.9 (2)	C23B—Fe1B—C17B—C16B	145.3 (2)
Ga1A—Fe1A—C17A—C16A	41.17 (17)	Ga1B—Fe1B—C17B—C16B	39.34 (18)
C16A—C17A—C18A—C19A	−1.2 (4)	C16B—C17B—C18B—C19B	−0.8 (4)
Fe1A—C17A—C18A—C19A	−60.0 (3)	Fe1B—C17B—C18B—C19B	−59.8 (3)
C16A—C17A—C18A—Fe1A	58.8 (3)	C16B—C17B—C18B—Fe1B	58.9 (3)
C17A—Fe1A—C18A—C19A	119.1 (3)	C22B—Fe1B—C18B—C19B	−177.0 (2)
C22A—Fe1A—C18A—C19A	−172.8 (2)	C17B—Fe1B—C18B—C19B	119.2 (3)
C25A—Fe1A—C18A—C19A	−16.5 (9)	C25B—Fe1B—C18B—C19B	−26.1 (8)
C16A—Fe1A—C18A—C19A	82.0 (2)	C20B—Fe1B—C18B—C19B	37.3 (2)
C20A—Fe1A—C18A—C19A	37.5 (2)	C21B—Fe1B—C18B—C19B	134.9 (3)
C21A—Fe1A—C18A—C19A	139.4 (3)	C16B—Fe1B—C18B—C19B	82.1 (2)
C23A—Fe1A—C18A—C19A	−126.5 (2)	C24B—Fe1B—C18B—C19B	−86.6 (3)
C24A—Fe1A—C18A—C19A	−82.5 (3)	C23B—Fe1B—C18B—C19B	−130.8 (2)
Ga1A—Fe1A—C18A—C19A	101.0 (2)	Ga1B—Fe1B—C18B—C19B	98.8 (2)
C22A—Fe1A—C18A—C17A	68.1 (3)	C22B—Fe1B—C18B—C17B	63.8 (3)
C25A—Fe1A—C18A—C17A	−135.6 (8)	C25B—Fe1B—C18B—C17B	−145.3 (6)
C16A—Fe1A—C18A—C17A	−37.2 (2)	C20B—Fe1B—C18B—C17B	−81.9 (2)
C20A—Fe1A—C18A—C17A	−81.6 (3)	C21B—Fe1B—C18B—C17B	15.6 (4)
C21A—Fe1A—C18A—C17A	20.3 (4)	C16B—Fe1B—C18B—C17B	−37.2 (2)
C23A—Fe1A—C18A—C17A	114.4 (3)	C19B—Fe1B—C18B—C17B	−119.2 (3)
C24A—Fe1A—C18A—C17A	158.4 (2)	C24B—Fe1B—C18B—C17B	154.2 (2)
C19A—Fe1A—C18A—C17A	−119.1 (3)	C23B—Fe1B—C18B—C17B	110.0 (2)
Ga1A—Fe1A—C18A—C17A	−18.1 (3)	Ga1B—Fe1B—C18B—C17B	−20.4 (3)
C17A—C18A—C19A—C20A	0.6 (4)	C17B—C18B—C19B—C20B	0.6 (4)
Fe1A—C18A—C19A—C20A	−58.0 (2)	Fe1B—C18B—C19B—C20B	−58.0 (2)
C17A—C18A—C19A—Fe1A	58.6 (3)	C17B—C18B—C19B—Fe1B	58.6 (3)
C17A—Fe1A—C19A—C18A	−38.0 (2)	C22B—Fe1B—C19B—C18B	8.7 (6)
C22A—Fe1A—C19A—C18A	18.9 (6)	C17B—Fe1B—C19B—C18B	−38.0 (2)
C25A—Fe1A—C19A—C18A	176.1 (2)	C25B—Fe1B—C19B—C18B	172.9 (2)
C16A—Fe1A—C19A—C18A	−82.8 (2)	C20B—Fe1B—C19B—C18B	−120.1 (3)
C20A—Fe1A—C19A—C18A	−119.7 (3)	C21B—Fe1B—C19B—C18B	−130.7 (3)
C21A—Fe1A—C19A—C18A	−125.7 (4)	C16B—Fe1B—C19B—C18B	−82.6 (2)
C23A—Fe1A—C19A—C18A	79.9 (3)	C24B—Fe1B—C19B—C18B	124.2 (2)
C24A—Fe1A—C19A—C18A	127.5 (2)	C23B—Fe1B—C19B—C18B	76.0 (3)
Ga1A—Fe1A—C19A—C18A	−96.5 (2)	Ga1B—Fe1B—C19B—C18B	−98.6 (2)
C17A—Fe1A—C19A—C20A	81.7 (2)	C22B—Fe1B—C19B—C20B	128.8 (5)
C22A—Fe1A—C19A—C20A	138.6 (4)	C17B—Fe1B—C19B—C20B	82.0 (2)
C25A—Fe1A—C19A—C20A	−64.2 (3)	C25B—Fe1B—C19B—C20B	−67.0 (3)
C16A—Fe1A—C19A—C20A	36.9 (2)	C21B—Fe1B—C19B—C20B	−10.6 (4)
C21A—Fe1A—C19A—C20A	−6.0 (5)	C16B—Fe1B—C19B—C20B	37.4 (2)
C18A—Fe1A—C19A—C20A	119.7 (3)	C18B—Fe1B—C19B—C20B	120.1 (3)
C23A—Fe1A—C19A—C20A	−160.3 (2)	C24B—Fe1B—C19B—C20B	−115.7 (2)
C24A—Fe1A—C19A—C20A	−112.8 (2)	C23B—Fe1B—C19B—C20B	−163.9 (2)
Ga1A—Fe1A—C19A—C20A	23.3 (3)	Ga1B—Fe1B—C19B—C20B	21.4 (2)
C18A—C19A—C20A—C16A	0.2 (4)	C18B—C19B—C20B—C16B	−0.1 (4)
Fe1A—C19A—C20A—C16A	−58.2 (2)	Fe1B—C19B—C20B—C16B	−58.7 (2)
C18A—C19A—C20A—Fe1A	58.4 (3)	C18B—C19B—C20B—Fe1B	58.5 (3)
C17A—C16A—C20A—C19A	−0.9 (4)	C17B—C16B—C20B—C19B	−0.4 (4)
Ga1A—C16A—C20A—C19A	132.7 (3)	Ga1B—C16B—C20B—C19B	134.3 (3)
Fe1A—C16A—C20A—C19A	59.1 (3)	Fe1B—C16B—C20B—C19B	59.4 (2)
C17A—C16A—C20A—Fe1A	−60.1 (2)	C17B—C16B—C20B—Fe1B	−59.8 (2)
Ga1A—C16A—C20A—Fe1A	73.5 (2)	Ga1B—C16B—C20B—Fe1B	74.9 (2)
C17A—Fe1A—C20A—C19A	−81.2 (2)	C22B—Fe1B—C20B—C19B	−154.8 (3)
C22A—Fe1A—C20A—C19A	−153.1 (3)	C17B—Fe1B—C20B—C19B	−81.0 (2)
C25A—Fe1A—C20A—C19A	134.0 (2)	C25B—Fe1B—C20B—C19B	131.3 (2)
C16A—Fe1A—C20A—C19A	−121.5 (3)	C21B—Fe1B—C20B—C19B	174.8 (2)
C21A—Fe1A—C20A—C19A	177.3 (2)	C16B—Fe1B—C20B—C19B	−121.0 (3)
C18A—Fe1A—C20A—C19A	−37.0 (2)	C18B—Fe1B—C20B—C19B	−36.9 (2)
C23A—Fe1A—C20A—C19A	71.5 (6)	C24B—Fe1B—C20B—C19B	93.5 (2)
C24A—Fe1A—C20A—C19A	94.4 (3)	C23B—Fe1B—C20B—C19B	75.4 (8)
Ga1A—Fe1A—C20A—C19A	−158.1 (2)	Ga1B—Fe1B—C20B—C19B	−159.6 (2)
C17A—Fe1A—C20A—C16A	40.3 (2)	C22B—Fe1B—C20B—C16B	−33.8 (4)
C22A—Fe1A—C20A—C16A	−31.6 (4)	C17B—Fe1B—C20B—C16B	40.0 (2)
C25A—Fe1A—C20A—C16A	−104.5 (2)	C25B—Fe1B—C20B—C16B	−107.7 (2)
C21A—Fe1A—C20A—C16A	−61.3 (2)	C21B—Fe1B—C20B—C16B	−64.2 (2)
C18A—Fe1A—C20A—C16A	84.4 (2)	C18B—Fe1B—C20B—C16B	84.1 (2)
C23A—Fe1A—C20A—C16A	−167.1 (5)	C19B—Fe1B—C20B—C16B	121.0 (3)
C24A—Fe1A—C20A—C16A	−144.1 (2)	C24B—Fe1B—C20B—C16B	−145.5 (2)
C19A—Fe1A—C20A—C16A	121.5 (3)	C23B—Fe1B—C20B—C16B	−163.6 (7)
Ga1A—Fe1A—C20A—C16A	−36.58 (18)	Ga1B—Fe1B—C20B—C16B	−38.55 (17)
C16A—Ga1A—C21A—C25A	65.7 (3)	C16B—Ga1B—C21B—C22B	−64.1 (3)
C7A—Ga1A—C21A—C25A	−152.9 (2)	C7B—Ga1B—C21B—C22B	77.1 (3)
N1A—Ga1A—C21A—C25A	−44.2 (3)	N1b—Ga1B—C21B—C22B	−169.6 (2)
Fe1A—Ga1A—C21A—C25A	64.6 (2)	Fe1B—Ga1B—C21B—C22B	−65.1 (2)
C16A—Ga1A—C21A—C22A	−62.0 (3)	C16B—Ga1B—C21B—C25B	65.4 (3)
C7A—Ga1A—C21A—C22A	79.5 (3)	C7B—Ga1B—C21B—C25B	−153.4 (3)
N1A—Ga1A—C21A—C22A	−171.9 (2)	N1b—Ga1B—C21B—C25B	−40.1 (3)
Fe1A—Ga1A—C21A—C22A	−63.1 (2)	Fe1B—Ga1B—C21B—C25B	64.4 (2)
C16A—Ga1A—C21A—Fe1A	1.07 (14)	C16B—Ga1B—C21B—Fe1B	1.02 (14)
C7A—Ga1A—C21A—Fe1A	142.53 (13)	C7B—Ga1B—C21B—Fe1B	142.22 (13)
N1A—Ga1A—C21A—Fe1A	−108.82 (12)	N1b—Ga1B—C21B—Fe1B	−104.47 (12)
C17A—Fe1A—C21A—C25A	−159.6 (2)	C17B—Fe1B—C21B—C22B	81.4 (2)
C22A—Fe1A—C21A—C25A	115.3 (3)	C25B—Fe1B—C21B—C22B	−115.7 (3)
C16A—Fe1A—C21A—C25A	−121.0 (2)	C20B—Fe1B—C21B—C22B	155.9 (2)
C20A—Fe1A—C21A—C25A	−85.5 (2)	C16B—Fe1B—C21B—C22B	119.1 (2)
C18A—Fe1A—C21A—C25A	−173.4 (3)	C18B—Fe1B—C21B—C22B	70.6 (4)
C23A—Fe1A—C21A—C25A	79.0 (2)	C19B—Fe1B—C21B—C22B	163.3 (3)
C24A—Fe1A—C21A—C25A	36.2 (2)	C24B—Fe1B—C21B—C22B	−79.2 (2)
C19A—Fe1A—C21A—C25A	−81.3 (4)	C23B—Fe1B—C21B—C22B	−36.3 (2)
Ga1A—Fe1A—C21A—C25A	−119.9 (2)	Ga1B—Fe1B—C21B—C22B	120.1 (2)
C17A—Fe1A—C21A—C22A	85.1 (2)	C22B—Fe1B—C21B—C25B	115.7 (3)
C25A—Fe1A—C21A—C22A	−115.3 (3)	C17B—Fe1B—C21B—C25B	−162.9 (2)
C16A—Fe1A—C21A—C22A	123.7 (2)	C20B—Fe1B—C21B—C25B	−88.5 (2)
C20A—Fe1A—C21A—C22A	159.2 (2)	C16B—Fe1B—C21B—C25B	−125.3 (2)
C18A—Fe1A—C21A—C22A	71.2 (4)	C18B—Fe1B—C21B—C25B	−173.8 (3)
C23A—Fe1A—C21A—C22A	−36.3 (2)	C19B—Fe1B—C21B—C25B	−81.0 (3)
C24A—Fe1A—C21A—C22A	−79.1 (2)	C24B—Fe1B—C21B—C25B	36.5 (2)
C19A—Fe1A—C21A—C22A	163.4 (3)	C23B—Fe1B—C21B—C25B	79.3 (2)
Ga1A—Fe1A—C21A—C22A	124.7 (2)	Ga1B—Fe1B—C21B—C25B	−124.3 (2)
C17A—Fe1A—C21A—Ga1A	−39.65 (17)	C22B—Fe1B—C21B—Ga1B	−120.1 (2)
C22A—Fe1A—C21A—Ga1A	−124.7 (2)	C17B—Fe1B—C21B—Ga1B	−38.62 (18)
C25A—Fe1A—C21A—Ga1A	119.9 (2)	C25B—Fe1B—C21B—Ga1B	124.3 (2)
C16A—Fe1A—C21A—Ga1A	−1.06 (14)	C20B—Fe1B—C21B—Ga1B	35.82 (17)
C20A—Fe1A—C21A—Ga1A	34.42 (18)	C16B—Fe1B—C21B—Ga1B	−1.01 (14)
C18A—Fe1A—C21A—Ga1A	−53.5 (3)	C18B—Fe1B—C21B—Ga1B	−49.5 (4)
C23A—Fe1A—C21A—Ga1A	−161.04 (17)	C19B—Fe1B—C21B—Ga1B	43.3 (3)
C24A—Fe1A—C21A—Ga1A	156.17 (17)	C24B—Fe1B—C21B—Ga1B	160.77 (17)
C19A—Fe1A—C21A—Ga1A	38.7 (4)	C23B—Fe1B—C21B—Ga1B	−156.39 (17)
C25A—C21A—C22A—C23A	−0.7 (4)	C25B—C21B—C22B—C23B	−0.2 (4)
Ga1A—C21A—C22A—C23A	132.6 (3)	Ga1B—C21B—C22B—C23B	136.8 (3)
Fe1A—C21A—C22A—C23A	59.3 (2)	Fe1B—C21B—C22B—C23B	60.2 (3)
C25A—C21A—C22A—Fe1A	−60.0 (2)	C25B—C21B—C22B—Fe1B	−60.4 (2)
Ga1A—C21A—C22A—Fe1A	73.3 (2)	Ga1B—C21B—C22B—Fe1B	76.6 (2)
C17A—Fe1A—C22A—C23A	132.3 (2)	C17B—Fe1B—C22B—C23B	128.7 (2)
C25A—Fe1A—C22A—C23A	−81.2 (2)	C25B—Fe1B—C22B—C23B	−81.3 (2)
C16A—Fe1A—C22A—C23A	174.2 (2)	C20B—Fe1B—C22B—C23B	−166.3 (3)
C20A—Fe1A—C22A—C23A	−163.7 (3)	C21B—Fe1B—C22B—C23B	−121.3 (3)
C21A—Fe1A—C22A—C23A	−121.3 (3)	C16B—Fe1B—C22B—C23B	170.6 (2)
C18A—Fe1A—C22A—C23A	94.2 (2)	C18B—Fe1B—C22B—C23B	92.1 (2)
C24A—Fe1A—C22A—C23A	−37.1 (2)	C19B—Fe1B—C22B—C23B	85.4 (5)
C19A—Fe1A—C22A—C23A	80.0 (5)	C24B—Fe1B—C22B—C23B	−37.5 (2)
Ga1A—Fe1A—C22A—C23A	−158.7 (2)	Ga1B—Fe1B—C22B—C23B	−161.5 (2)
C17A—Fe1A—C22A—C21A	−106.3 (2)	C17B—Fe1B—C22B—C21B	−110.0 (2)
C25A—Fe1A—C22A—C21A	40.13 (19)	C25B—Fe1B—C22B—C21B	40.01 (19)
C16A—Fe1A—C22A—C21A	−64.5 (2)	C20B—Fe1B—C22B—C21B	−45.0 (3)
C20A—Fe1A—C22A—C21A	−42.4 (4)	C16B—Fe1B—C22B—C21B	−68.1 (2)
C18A—Fe1A—C22A—C21A	−144.5 (2)	C18B—Fe1B—C22B—C21B	−146.6 (2)
C23A—Fe1A—C22A—C21A	121.3 (3)	C19B—Fe1B—C22B—C21B	−153.3 (4)
C24A—Fe1A—C22A—C21A	84.3 (2)	C24B—Fe1B—C22B—C21B	83.8 (2)
C19A—Fe1A—C22A—C21A	−158.6 (4)	C23B—Fe1B—C22B—C21B	121.3 (3)
Ga1A—Fe1A—C22A—C21A	−37.43 (17)	Ga1B—Fe1B—C22B—C21B	−40.19 (17)
C21A—C22A—C23A—C24A	0.4 (4)	C21B—C22B—C23B—C24B	0.0 (4)
Fe1A—C22A—C23A—C24A	59.1 (2)	Fe1B—C22B—C23B—C24B	59.1 (3)
C21A—C22A—C23A—Fe1A	−58.7 (2)	C21B—C22B—C23B—Fe1B	−59.1 (2)
C17A—Fe1A—C23A—C24A	177.8 (2)	C22B—Fe1B—C23B—C24B	−118.9 (3)
C22A—Fe1A—C23A—C24A	−119.8 (3)	C17B—Fe1B—C23B—C24B	176.0 (2)
C25A—Fe1A—C23A—C24A	−38.0 (2)	C25B—Fe1B—C23B—C24B	−37.3 (2)
C16A—Fe1A—C23A—C24A	−131.7 (3)	C20B—Fe1B—C23B—C24B	22.1 (8)
C20A—Fe1A—C23A—C24A	29.2 (6)	C21B—Fe1B—C23B—C24B	−81.8 (2)
C21A—Fe1A—C23A—C24A	−82.5 (2)	C16B—Fe1B—C23B—C24B	−136.4 (3)
C18A—Fe1A—C23A—C24A	131.2 (2)	C18B—Fe1B—C23B—C24B	129.5 (2)
C19A—Fe1A—C23A—C24A	86.4 (3)	C19B—Fe1B—C23B—C24B	85.6 (3)
Ga1A—Fe1A—C23A—C24A	−97.2 (2)	Ga1B—Fe1B—C23B—C24B	−99.75 (19)
C17A—Fe1A—C23A—C22A	−62.4 (3)	C17B—Fe1B—C23B—C22B	−65.1 (3)
C25A—Fe1A—C23A—C22A	81.8 (2)	C25B—Fe1B—C23B—C22B	81.6 (2)
C16A—Fe1A—C23A—C22A	−11.9 (4)	C20B—Fe1B—C23B—C22B	140.9 (7)
C20A—Fe1A—C23A—C22A	149.0 (5)	C21B—Fe1B—C23B—C22B	37.1 (2)
C21A—Fe1A—C23A—C22A	37.3 (2)	C16B—Fe1B—C23B—C22B	−17.5 (4)
C18A—Fe1A—C23A—C22A	−109.0 (2)	C18B—Fe1B—C23B—C22B	−111.7 (2)
C24A—Fe1A—C23A—C22A	119.8 (3)	C19B—Fe1B—C23B—C22B	−155.6 (2)
C19A—Fe1A—C23A—C22A	−153.9 (2)	C24B—Fe1B—C23B—C22B	118.9 (3)
Ga1A—Fe1A—C23A—C22A	22.5 (2)	Ga1B—Fe1B—C23B—C22B	19.1 (2)
C22A—C23A—C24A—C25A	0.2 (4)	C22B—C23B—C24B—C25B	0.2 (4)
Fe1A—C23A—C24A—C25A	58.4 (2)	Fe1B—C23B—C24B—C25B	58.1 (2)
C22A—C23A—C24A—Fe1A	−58.2 (2)	C22B—C23B—C24B—Fe1B	−57.9 (2)
C17A—Fe1A—C24A—C23A	−6.9 (6)	C22B—Fe1B—C24B—C25B	−82.0 (2)
C22A—Fe1A—C24A—C23A	37.5 (2)	C17B—Fe1B—C24B—C25B	−130.9 (4)
C25A—Fe1A—C24A—C23A	119.1 (3)	C20B—Fe1B—C24B—C25B	65.6 (3)
C16A—Fe1A—C24A—C23A	135.6 (3)	C21B—Fe1B—C24B—C25B	−37.4 (2)
C20A—Fe1A—C24A—C23A	−170.9 (2)	C16B—Fe1B—C24B—C25B	7.4 (4)
C21A—Fe1A—C24A—C23A	82.4 (2)	C18B—Fe1B—C24B—C25B	162.8 (2)
C18A—Fe1A—C24A—C23A	−75.9 (3)	C19B—Fe1B—C24B—C25B	114.5 (2)
C19A—Fe1A—C24A—C23A	−122.9 (2)	C23B—Fe1B—C24B—C25B	−120.0 (3)
Ga1A—Fe1A—C24A—C23A	100.6 (2)	Ga1B—Fe1B—C24B—C25B	−22.7 (2)
C17A—Fe1A—C24A—C25A	−126.1 (5)	C22B—Fe1B—C24B—C23B	38.0 (2)
C22A—Fe1A—C24A—C25A	−81.6 (2)	C17B—Fe1B—C24B—C23B	−10.9 (6)
C16A—Fe1A—C24A—C25A	16.5 (4)	C25B—Fe1B—C24B—C23B	120.0 (3)
C20A—Fe1A—C24A—C25A	69.9 (2)	C20B—Fe1B—C24B—C23B	−174.4 (2)
C21A—Fe1A—C24A—C25A	−36.8 (2)	C21B—Fe1B—C24B—C23B	82.6 (2)
C18A—Fe1A—C24A—C25A	165.0 (2)	C16B—Fe1B—C24B—C23B	127.4 (3)
C23A—Fe1A—C24A—C25A	−119.1 (3)	C18B—Fe1B—C24B—C23B	−77.2 (3)
C19A—Fe1A—C24A—C25A	118.0 (2)	C19B—Fe1B—C24B—C23B	−125.5 (2)
Ga1A—Fe1A—C24A—C25A	−18.5 (2)	Ga1B—Fe1B—C24B—C23B	97.3 (2)
C23A—C24A—C25A—C21A	−0.7 (4)	C23B—C24B—C25B—C21B	−0.4 (4)
Fe1A—C24A—C25A—C21A	58.6 (2)	Fe1B—C24B—C25B—C21B	58.8 (2)
C23A—C24A—C25A—Fe1A	−59.3 (2)	C23B—C24B—C25B—Fe1B	−59.2 (3)
C22A—C21A—C25A—C24A	0.9 (4)	C22B—C21B—C25B—C24B	0.4 (4)
Ga1A—C21A—C25A—C24A	−134.9 (3)	Ga1B—C21B—C25B—C24B	−134.5 (3)
Fe1A—C21A—C25A—C24A	−59.0 (2)	Fe1B—C21B—C25B—C24B	−59.8 (2)
C22A—C21A—C25A—Fe1A	59.9 (2)	C22B—C21B—C25B—Fe1B	60.2 (2)
Ga1A—C21A—C25A—Fe1A	−75.82 (19)	Ga1B—C21B—C25B—Fe1B	−74.7 (2)
C17A—Fe1A—C25A—C24A	158.0 (3)	C22B—Fe1B—C25B—C24B	80.8 (2)
C22A—Fe1A—C25A—C24A	81.3 (2)	C17B—Fe1B—C25B—C24B	152.9 (3)
C16A—Fe1A—C25A—C24A	−171.6 (2)	C20B—Fe1B—C25B—C24B	−132.7 (2)
C20A—Fe1A—C25A—C24A	−128.2 (2)	C21B—Fe1B—C25B—C24B	120.8 (3)
C21A—Fe1A—C25A—C24A	121.7 (3)	C16B—Fe1B—C25B—C24B	−176.4 (2)
C18A—Fe1A—C25A—C24A	−77.1 (9)	C18B—Fe1B—C25B—C24B	−73.2 (7)
C23A—Fe1A—C25A—C24A	37.4 (2)	C19B—Fe1B—C25B—C24B	−94.3 (3)
C19A—Fe1A—C25A—C24A	−90.8 (3)	C23B—Fe1B—C25B—C24B	36.9 (2)
Ga1A—Fe1A—C25A—C24A	162.1 (2)	Ga1B—Fe1B—C25B—C24B	158.4 (2)
C17A—Fe1A—C25A—C21A	36.3 (4)	C22B—Fe1B—C25B—C21B	−40.0 (2)
C22A—Fe1A—C25A—C21A	−40.4 (2)	C17B—Fe1B—C25B—C21B	32.2 (4)
C16A—Fe1A—C25A—C21A	66.7 (2)	C20B—Fe1B—C25B—C21B	106.5 (2)
C20A—Fe1A—C25A—C21A	110.0 (2)	C16B—Fe1B—C25B—C21B	62.8 (2)
C18A—Fe1A—C25A—C21A	161.2 (8)	C18B—Fe1B—C25B—C21B	166.0 (6)
C23A—Fe1A—C25A—C21A	−84.4 (2)	C19B—Fe1B—C25B—C21B	144.9 (2)
C24A—Fe1A—C25A—C21A	−121.7 (3)	C24B—Fe1B—C25B—C21B	−120.8 (3)
C19A—Fe1A—C25A—C21A	147.5 (2)	C23B—Fe1B—C25B—C21B	−83.8 (2)
Ga1A—Fe1A—C25A—C21A	40.39 (17)	Ga1B—Fe1B—C25B—C21B	37.64 (17)
